# 1,2,4,5‐Tetrazine‐Boron Halide Adduct Formation Unraveled in Solution and Solid State

**DOI:** 10.1002/open.70317

**Published:** 2026-10-01

**Authors:** Jona Voss, Andreas Mix, Jan‐Hendrik Lamm, Beate Neumann, Hans‐Georg Stammler, Norbert Sewald

**Affiliations:** ^1^ Organic and Bioorganic Chemistry Bielefeld University Bielefeld Germany; ^2^ Lehrstuhl für Anorganische Chemie und Strukturchemie Bielefeld University Bielefeld Germany

**Keywords:** DOSY NMR, Lewis acids, Lewis bases, N ligands, X‐ray diffraction

## Abstract

1,2,4,5‐Tetrazines are electron‐deficient heterocycles with four nitrogen atom‐localized lone pairs potentially acting as Lewis bases. While it has been shown that tetrazines, as electronically amphiphilic ligands, undergo anion–π, π–π, and transition metal‐based adduct formation reactions, adducts of tetrazines with main group metal Lewis acids have not yet been structurally characterized. We report spectroscopic and single crystal X‐ray diffraction (SC‐XRD) analyses of tetrazine‐boron halide adducts in solution and in the solid state, respectively. With the aid of (diffusion‐ordered) nuclear magnetic resonance (DOSY‐)NMR spectroscopy dynamic equilibria between tetrazine‐boron halide adducts have been proven, while solid state structures show localized 1:1 tetrazine‐boron halide adducts.

## Graphical abstract

1,2,4,5‐Tetrazines form Lewis adducts with boron halides BX_3_ (X = Cl, Br), a process being visually observable by a vibrant color change from pink to red. In the solid state, localized 1:1 tetrazine‐BX_3_ adducts are present. In contrast, ^1^H (DOSY‐)NMR spectroscopic titration revealed dynamic equilibria between even over‐stoichiometric tetrazine‐BX_3_ adducts, corroborated by comparison of volumes derived theoretically, from solution and solid state.

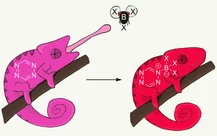

## 1 Introduction

1,2,4,5‐Tetrazines emerge as multifaceted building blocks in modern chemistry, essentially due to their four sp^2^ configured nitrogen atoms present in their aromatic ring and, subsequently, their energetically low π*‐type lowest unoccupied molecular orbital (LUMO) [1], making them suitable substrates for inverse electron demand Diels–Alder reactions (IEDDA) [2], anion–π‐ [3] and π–π‐interacting [4, 5, 6] adduct formation reactions, but also prone to reduction to radical anions and dihydrotetrazines [7, 8, 9]. Contrary, tetrazines exhibit an amphiphilic electronic situation due to their four nitrogen‐centered lone pairs, being able to act as Lewis bases as demonstrated by intermolecular tetrazine N⋯H contacts in the solid state [4, 10, 11] and azaphilic additions to tetrazines [12, 13, 14], particularly the formation of tetrazine‐transition metal complexes [1, 15, 16]. Generally, tetrazines are very weak bases with the conjugate acid having a p*K*_a_ < 0 [16, 17, 18], giving rise to unstable adducts. However, furnishing tetrazines with donor group containing substituents in 3‐ and 6‐position, such as 2‐pyridinyl or 2‐pyrimidinyl [15, 19] stabilizes these adducts. While the field of tetrazine‐transition metal adducts has been pioneered by Kaim and others [1, 15], tetrazine‐main group element adducts remain scarcely touched. Interestingly, there is only one report on a tetrazine‐main group element adduct that can be considered, tetrazine‐borane: In 2025, Zhu and colleagues reported on intramolecularly difluoroboronated tetrazines, possessing BF_2_ groups coordinated by a tetrazine nitrogen atom and an oxygen atom of a pendant 2‐oxyphenyl substituent (Scheme 1a) [20]. Additionally, Wegner and colleagues postulate the formation of a catalytically active intermolecular tetrazine‐bisborane complex, which, however, could not be characterized [21]. It has recently been shown that the reaction between a bis(pyrazolyl)tetrazine and two equivalents of bis(pentafluorophenyl)borane yields a 1:2 adduct; however, the tetrazine moiety was reduced to a 1,4‐dihydrotetrazine motif under oxidative H_2_ release from the borane [22]. Further, the addition of tetrazine to a dialkylgallium moiety by cooperative action of a Lewis basic phosphane moiety has been shown [23]. We herein report on the characterization of Lewis acid base adducts between 3,6‐diphenyl‐1,2,4,5‐tetrazine and boron halides in solution and solid state by combination of UV‐Vis and diffusion‐ordered nuclear magnetic resonance (DOSY‐NMR) spectroscopic and single‐crystal X‐ray diffraction (SC‐XRD) analyses.

**SCHEME 1:**
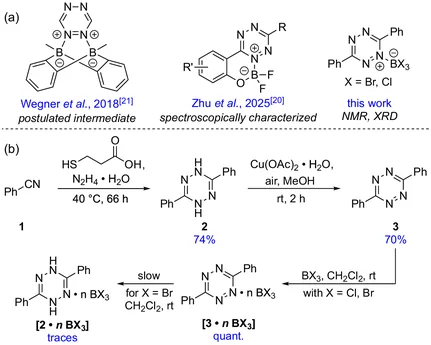
(a) Tetrazine‐borane adducts reported in the literature [20, 21] (R = alkyl, alkenyl, aryl; R′ = H, Me, OMe, Br) and adducts discussed in this work and (b) synthesis of tetrazine **3** from benzonitrile **1** and adduct formation reactions with boron halides.

## 2 Results and Discussion

In 2019 Wombacher and colleagues might have given the first hints to the presence of a tetrazine‐boron adduct, attempting a methyl deprotection of a (methoxymethyl)tetrazine with BBr_3_, during which the tetrazine’s typical reddish‐pink color changed to orange [24]. Addition of methanol after completion of the reaction re‐established the tetrazine’s parent color, hinting to the labile nature of the putatively formed adduct. Eager to elaborate on this phenomenon, we investigated the reaction between structurally simple 3,6‐diphenyl‐1,2,4,5‐tetrazine (TzPh_2_, **3**) [25] and boron halides.

Thiol‐catalyzed condensation between benzonitrile **1** and hydrazine hydrate affords 1,4‐dihydrotetrazine (*d*TzPh_2_, **2**), which was structurally characterized by SC‐XRD (see below and Supporting Information), and is readily oxidized by Cu^II^‐catalysis with air to obtain tetrazine **3** (Scheme 1b).

Immediate color change from pink to orange was observed after addition of BCl_3_ and BBr_3_ to a solution of tetrazine **3** in CH_2_Cl_2_, being in line with Wombacher’s report [24]. UV–Vis spectroscopic analyses of the obtained solutions (Figure 1, left) revealed hypsochromic shifts of the tetrazine’s parent absorption band at 547 to 498 nm (**3** • *n* BCl_3_) and to 506 nm (**3** • *n* BBr_3_), respectively, matching the observed color change from pink to orange [26].

**FIGURE 1:**
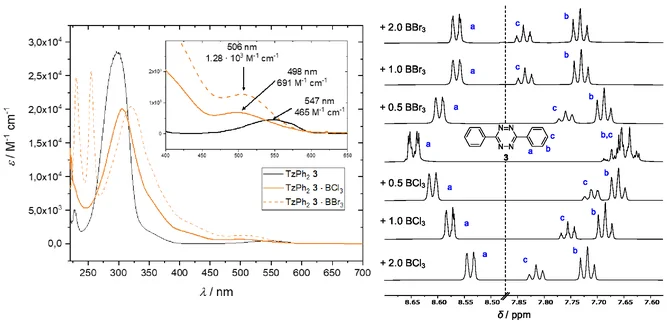
Left: UV–Vis spectra of TzPh_2_ **3** and the adducts TzPh_2_ **3** • BX_3_ (X = Cl, Br) in dichloromethane (the spectra were virtually similar for *n* = 1, 2.5); right: expanded regions of ^1^H NMR spectra (600 MHz, 293 K, dichloromethane‐*d*_2_) of TzPh_2_ **3** with different equivalents *n* of BCl_3_ and BBr_3_ (signal sets remain virtually isochronous with more equivalents of BX_3_).

^1^H NMR spectroscopic investigation of mixtures of tetrazine **3** and BX_3_ (X = F, Cl, Br, I) showed different findings: While addition of the weakest Lewis acid BF_3_ to tetrazine **3** caused no significant shift of the signals, mixtures with BCl_3_ and most notably with BBr_3_ caused shifts of all observable signals hinting to adduct formation. Addition of BI_3_ caused immediate and complete degradation of tetrazine **3**. Clearly, the association process of tetrazine with BX_3_ correlates with the Lewis acidity of boron trihalides increasing in the order BF_3_ < BCl_3_ < BBr_3_ < BI_3_ [27, 28, 29]. Looking more closely at the ^1^H NMR spectra of tetrazine **3** + *n* BX_3_ (with *n* = 0–5.0 and X = Cl, Br; Figure 1, right) two major findings can be observed: First, spectra of tetrazine **3** + 0.5 BX_3_ show single signal sets for the protons of both phenyl substituents, indicative for dynamic B⋯N contacts between the adjacent N atoms. Second, for tetrazine **3** + *n* BBr_3_ from *n* = 0 to 1.0 the signals shift toward each other, while only showing a small shift up to *n* = 2.0 and remaining virtually isochronous for *n* = 2–5, indicating that tetrazine **3** is nearly coordinatively saturated with one equivalent of BBr_3_. Contrary, tetrazine **3** seems to be coordinatively saturated only with at least two equivalents of BCl_3_ as indicated by merging signals from *n* = 0 to 2.0. Upon addition of methanol to mixtures of tetrazine **3** and BCl_3_ or BBr_3_, the tetrazine’s parent pink color is retained and re‐isolation of tetrazine **3** can be confirmed by NMR analyses.

The adduct formation reactions of TzPh_2_ **3** with BX_3_ (X = Cl, Br) were also monitored by ^1^H diffusion ordered (DOSY‐)NMR spectroscopy (Figure 2). Titration of TzPh_2_ **3** with up to 5.0 equivalents of BX_3_ (X = Cl, Br) confirmed the trends observed from the ^1^H NMR spectra. A comparison with the solvodynamic volume of free tetrazine, *V*_s_(TzPh_2_ **3**) = 262 ± 8 Å^3^, shows that *V*_s_ of **3** + *n* BBr_3_ strictly increases up to *n* = 1 with 545 ± 16 Å^3^ and up to *n* = 2 with 469 ± 14 Å^3^ for **3** + *n* BCl_3_. Even though only small shifts in the ^1^H NMR spectra of tetrazine **3** + *n* BBr_3_ were observed with *n* > 1 (see Figure 1, right), DOSY analyses clearly show the presence of larger aggregates with *n* = 2 (*V*_s_ = 618 ± 19 Å^3^). Within the margins of error, solvodynamic volumes stagnate for both adducts with increasing *n* each (see also Supporting Information: Chapter 3).

**FIGURE 2:**
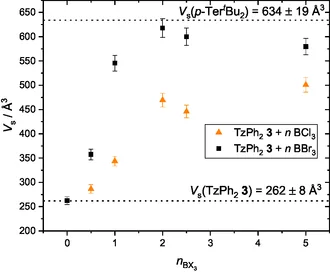
Solvodynamic volumes *V*_s_ of TzPh_2_ **3** + *n* BX_3_ (X = Cl, Br) with different equivalents of BX_3_ (*n* = 0–5.0) obtained from ^1^H DOSY‐NMR spectroscopic measurements with mean errors in dichloromethane‐*d*_2_ at 293 K (refer to Supporting Information: Chapter 3).

Notably, the largest solvodynamic volume *V*_s,max_ of **3** + *n* BBr_3_ still is about 32% larger than *V*_s,max_ of **3** + *n* BCl_3_ (both obtained with *n* = 2), even though BCl_3_ and BBr_3_ show similar van der Waals volumes *V*_vdW_ of 87.2 and 92.3 Å^3^ (Δ*V*_vdW_ = 5.1 Å^3^), respectively (see Supporting Information: Chapter 3). It is likely that the adduct **3** • *n* BBr_3_, is a significantly more polar species than **3** • *n* BCl_3_ [27, 28, 29] and possesses a larger solvation shell consisting of polar molecules such as BBr_3_ itself and dichloromethane. Even though *V*_s,max_(**3** + *n* BCl_3_) is only reached with *n* ≥ 2, three major indications prompt that the dominant species in solution still is a 1:1 adduct, **3** • BCl_3_: First, since both BCl_3_ and BBr_3_ have similar *V*_vdW_, but BBr_3_ is the stronger Lewis acid, the fact that tetrazine **3** already shows strong adduct formation with just one equivalent of BBr_3_ indicates that tetrazine **3** is only coordinatively monosaturated in the presence of an excess of BCl_3_ (*n* ≥ 2). Second, 2′,5′‐di‐*tert*‐butyl‐*p*‐terphenyl (*p*‐Ter^*t*^Bu_2_) was synthesized as reference compound resembling tetrazine **3** • 2 BX_3_ with *V*_vdW_ = 434 Å^3^ [compared with similar *V*_vdW_ of **3** • 2 BCl_3_ (415 Å^3^) and **3** • 2 BBr_3_ (425 Å^3^)] and *V*_s_ = 634 ± 19 Å^3^, which exceeds *V*_s,max_(**3** + *n* BCl_3_) by 35% (for more information see Tables S2 and S3). Given both matching *V*_vdW_ and *V*_s_, as well as the additionally observed homotopicity of the phenyl substituents from ^1^H NMR signals of TzPh_2_ **3** • 2 BX_3_ (X = Cl, Br, Figure 1, right), the symmetrically substituted *p*‐Ter^*t*^Bu_2_ (Figure 3) is likely to be a suitable model compound for 1:2 tetrazine‐BX_3_ adducts.

**FIGURE 3:**
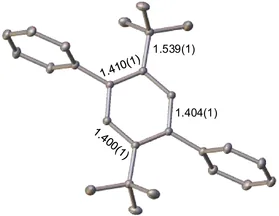
Molecular structure of *p*‐Ter^*t*^Bu_2_ as determined by SC‐XRD measurement with selected bond lengths (in Å). Thermal ellipsoids depicted at 50% probability. Hydrogen atoms were omitted for clarity. The omitted bond lengths of the central phenylene unit are generated by symmetry due to inversion at the ring centroid (symmetry code: 2 − *X*, 1 − *Y*, 1 − *Z*).

Strikingly, *V*_s_(*p*‐Ter^*t*^Bu_2_) = 634 ± 19 Å^3^ matches *V*_s_(**3** + 2 BBr_3_) = 618 ± 19 Å^3^, indicating that tetrazine **3** is capable of accommodating up to two equivalents of BBr_3_ but not of BCl_3_. Third, evidence for 1:1 adduct formation was given by SC‐XRD measurements of crystals obtained from concentrated *ortho*‐difluorobenzene solutions of tetrazine **3** with excesses of BCl_3_ and BBr_3_ (Figure 4) [30].

**FIGURE 4:**
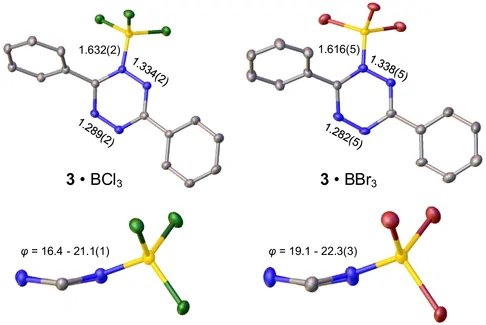
Molecular structures of TzPh_2_ **3** • BCl_3_ (left) and TzPh_2_ **3** • BBr_3_ (right) as determined by SC‐XRD measurements with selected bond lengths (in Å). Thermal ellipsoids depicted at 50% probability. Only one of the two molecules in the asymmetric unit of TzPh_2_ **3** • BCl_3_ and TzPh_2_ **3** • BBr_3_ is shown. Bottom: views along the C^aryl^–C^aryl^ axes of the tetrazine units with indicated out of plane angles *φ* (in °). Residual solvent molecules (for TzPh_2_ **3** • BBr_3_) and hydrogen atoms were omitted for clarity. The reported lengths match those described for the other molecules in the asymmetric units within a 3*σ* margin of error, respectively.

We would like to point out that for crystallization a greaseless Young tube should be used and all compounds should be handled under strict exclusion of both air and moisture to obtain crystals of suitable quality for SC‐XRD measurements. Obtained crystals of **3** • BX_3_ (X = Cl, Br) were transferred and selected under dry N_2_ at 240 K in cryogenic oil to prevent decomposition. Both 1:1 adducts, tetrazine **3** • BCl_3_ (*I*4_1_/*a*) and **3** • BBr_3_ (*I*2/*a*) crystallize with two crystallographically independent molecules in the asymmetric unit each, all of which show coordination of the BX_3_ moiety to a single N atom each with N⋯B distances *d*_NB_ of 1.632(2) and 1.616(5) Å for **3** • BCl_3_ and **3** • BBr_3_, respectively. The distance *d*_NB_ of **3** • BCl_3_ is slightly longer than for **3** • BBr_3_, but both are significantly longer than for the structural analogs pyridine • BCl_3_ [*d*_NB_ = 1.593(1) Å] [31] and pyridine • BBr_3_ [*d*_NB_ = 1.594(1) Å] [32], matching with tetrazine’s lower basicity compared with pyridine [17]. Comparison with free tetrazine **3** [25] shows that the bonding situation of the aromatic tetrazine core gets significantly altered upon adduct formation: While *d*_NN_ near to the BX_3_ moiety increases slightly (*d*_NN_(**3**) = 1.318(1) Å), *d*_NN_ distal to the BX_3_ moiety decreases significantly (cf. Figure 4), and all *d*_CN_ vary strongly between 1.321(5) and 1.376(5) Å (*d*_CN_(**3**) = 1.348(1) Å) [25], deforming the tetrazine core into a boat with out‐of‐plane angles of 16.4°–21.1(1)° and 19.1°–22.3(3)° for **3** • BCl_3_ and **3** • BBr_3_, respectively. The tetracoordinated boron atoms adopt nearly perfect tetragonal geometries with *τ*_4_ [33] = 0.968 (*τ*_4_′ [34] = 0.961) for **3** • BCl_3_ and *τ*_4_ = 0.979 (*τ*_4_′ = 0.968) for **3** • BBr_3_, respectively.

The volumes extracted from SC‐XRD measurements *V*_XRD_ of **3** • BCl_3_ (379 ± 1 Å^3^) and **3** • BBr_3_ (405 ± 1 Å^3^) are similar in magnitude, as expected from similar *V*_vdW_ (334 and 339 Å^3^, respectively) (Table 1). As mentioned above (Figure 2), the sizes differ in solution, likely due to a larger solvation shell of **3** • BBr_3_ (545 ± 19 Å^3^) as compared with **3** • BCl_3_ (344 ± 10 Å^3^). Even though ^1^H (DOSY‐)NMR data indicate that tetrazine **3** forms over‐stoichiometric adducts with excess of BBr_3_, only **3** • BBr_3_ crystallized from solution—attempts isolating **3** • 2 BBr_3_ as crystalline solid under various conditions failed, again hinting to the dynamic equilibria between different adducts in solution.

**TABLE 1:** Comparison of van der Waals volumes (*V*_vdW_) [35], volumes extracted from SC‐XRD measurements (*V*_XRD_), and of solvodynamic volumes (*V*_s_) of tetrazine **3** and its adducts **3** • BCl_3_ and **3** • BBr_3_. *V*_XRD_ were calculated by dividing the unit cell’s volumes by *Z*, after subtracting any volumes of cocrystallized solvent molecules with literature values (for more information refer to Supporting Information: Chapters 3 and 4).

	*V*_vdW_, Å^3^	*V*_XRD_, Å^3^	*V*_s_, Å^3^
TzPh_2_ **3**	253	273 ± 1	262 ± 8
TzPh_2_ **3** • BCl_3_	334	379 ± 1	344 ± 10^a^
TzPh_2_ **3** • BBr_3_	339	405 ± 1	545 ± 16^a^

a Data taken from measurements of tetrazine **3** with 1.0 equivalent of BX_3_ (X = Cl, Br) added.

During several attempts to obtain crystals of **3** • n BBr_3_ with suitable quality for SC‐XRD measurements, also crystals of reduced *d*TzPh_2_ **2** • 2 BBr_3_ were obtained from a solution of TzPh_2_ **3** and BBr_3_ after several days (Figure 5, bottom).

**FIGURE 5:**
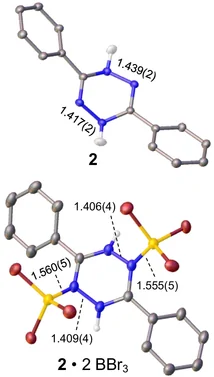
Molecular structures of *d*TzPh_2_ **2** (top) and *d*TzPh_2_ **2** • 2 BBr_3_‐α (bottom) as determined by SC‐XRD measurements with selected bond lengths (in Å). Thermal ellipsoids depicted at 50% probability. Residual solvent molecules (for *d*TzPh_2_ **2**) and hydrogen atoms attached to C atoms were omitted for clarity. Lengths and angles of *d*TzPh_2_ **2** • 2 BBr_3_‐α match those described for *d*TzPh_2_ **2** • 2 BBr_3_‐β within a 3*σ* margin of error (see Figure S5).

Authentic samples of *d*TzPh_2_ **2** • 2 BBr_3_ were also obtained from a mixture of *d*TzPh_2_ **2** and excess BBr_3_. The presence of the reduced 1,4‐dihydrotetrazine motif can be anticipated not only because of residual electron density at terminal N atoms (matching with H atoms) and the structural analogy to free *d*TzPh_2_ **2** (Figure 5, top), but also because of significantly shortened C⋯N distances (*d*_CN_ = 1.336(4) Å) of the C=N‐donating group as compared with TzPh_2_ **3** • BBr_3_ (*d*_CN_ = 1.359(5) Å) being in line with the expected higher basicity of the dihydro‐Tz over the Tz core [7]. Consequently, the N⋯B distances of *d*TzPh_2_ **2** • 2 BBr_3_ are significantly shorter (*d*_NB_ = 1.560(5) Å) than for TzPh_2_ **3** • BBr_3_ (*d*_NB_ = 1.616(5) Å).

The generation of *d*TzPh_2_ **2** from a mixture of TzPh_2_ **3** and BBr_3_ was unexpected and it could be shown by UV–Vis and NMR spectroscopic analyses that this reduction is a slow process with only about 10% of *d*TzPh_2_ **2** present after 3 days at room temperature (see Supporting Information: Chapter 6). With TzPh_2_ **3** being stable in solution and no observed formation of *d*TzPh_2_ **2** from a mixture of TzPh_2_ **3** and BCl_3_, it is hypothesized that due to Lewis acid coordination of BBr_3_ to tetrazine **3** its susceptibility to reduction is increased enough to be reduced to *d*TzPh_2_ **2**. Likely, due to the high tendency of hydrolysis, **3** • BBr_3_ is reduced by bromide ions, originating from hydrolysis of the BBr_3_ unit with trace water, a similar reduction of tetrazine **3** being described upon coordination with Sc^3+^ [36]. While solid state structures of free *d*TzPh_2_ **2** and *d*TzPh_2_ **2** • 2 BBr_3_ (Figure 5) show the presence of only 1,4‐dihydro isomers, (DOSY‐)NMR spectroscopic investigation of *d*TzPh_2_ **2** + *n* BBr_3_ indicate the presence of different isomers (see Supporting Information: Chapter 6, NMR spectrum 13).

## 3 Conclusion

1,2,4,5‐Tetrazines have been shown to act as Lewis bases forming adducts with boron halides BX_3_ of sufficient Lewis acidity (X = Cl, Br). While these adducts are of dynamic nature in solution as corroborated by ^1^H (DOSY‐)NMR titration, a comparison of molecular volumes derived theoretically, from solution, and from solid state indicates that tetrazine **3** forms 1:1 adducts with BCl_3_, **3** • BCl_3_, and is able to coordinate up to two equivalents of BBr_3_ forming **3** • 2 BBr_3_. With the aid of structures obtained from SC‐XRD measurements we report on the first structurally characterized tetrazine main group element adducts. These adducts were shown to be labile and highly prone to solvolysis, regenerating the parent tetrazine. For tetrazine **3** • *n* BBr_3_ the unprecedented reduction to the corresponding 1,4‐dihydrotetrazine **2** • 2 BBr_3_ was observed, a reaction that might be reasoned by reduction of the highly electron deficient adduct **3** • *n* BBr_3_ [36] through in situ formed bromide ions. Based on analyses of association processes of tetrazine‐boron halides in solution and their molecular structures, we demonstrate that tetrazines act as weak donors and form labile adducts with a reductive degradation pathway [36]; insights, which might pave the way toward the use of tetrazines in catalysis and the rational design of tetrazine‐complexes [15].

## Funding

This work was supported by the Studienstiftung des Deutschen Volkes.

## Conflicts of Interest

The authors declare no conflicts of interest.

## Supporting information

Supplementary Material

## Data Availability

The data that supports the findings of this study are available in the Supporting Information of this article.
