# Supplementary material for: 1,2,4,5‐Tetrazine‐Boron Halide Adduct Formation Unraveled in Solution and Solid State
Source: ChemistryOpen. 2026 Oct 1;15(10):e70317. doi: 10.1002/open.70317 (PMC13629465; doi:10.1002/open.70317)
Supplement: Supplementary file 1 [file OPEN-15-e70317-s001.pdf]

Supplementary Information

for

1,2,4,5-Tetrazine-Boron Halide Adduct Formation Unravelled in Solution and Solid State

Jona Voss, Andreas Mix, Jan-Hendrik Lamm, Beate Neumann, Hans-Georg Stammler, Norbert Sewald

Table of Contents

1 General 2
2 Experimental Procedures 2
3 ^{1}H DOSY NMR Spectroscopy 5
4 X-Ray Diffraction Data 8
5 UV-Vis Spectroscopy 14
6 NMR Spectra 15
7 References 34

1 General

All chemicals were purchased from commercial sources and used without further purification Dry solvents were obtained as described, stored and handled under dry argon atmosphere: dichloromethane (-d_2) [boiling with CaH_2, then distillation and storage over molecular sieve (3 Å)], *ortho*-difluorobenzene [storage over CaH_2, then distillation and storage over molecular sieve (3 Å)], pentane [storage over molecular sieve (3 Å)]. All operations were conducted by using Schlenk high vacuum systems with argon (Linde, argon 4.6, 99.996%) serving as an inert atmosphere, unless otherwise stated. Degassing of solutions by three consecutive cycles of freeze-pump-thaw. The temperatures given for the reactions refer to the bath temperature. ESI-MS measurements were performed using a Q-IMS-TOF mass spectrometer Synapt G2Si (Waters GmbH, Manchester, UK) in resolution mode, interfaced to a nano-ESI (internally calibrated with the protonated Leu-enkephalin ion). Determination of exact masses were performed using centroided data. NMR spectra were recorded on a Bruker Avance III 500 (Neo) or Bruker Avance III 600 instrument using the indicated deuterated solvent with residual protonated solvent signals[1] as internal standards at 293 K. UV-Vis spectroscopy was carried out on the single-beam spectrophotometer UV-3100PC (VWR). Optical system: single-beam grating 1200 lines mm^{-1} silicon photodetector; wavelength range: 190 – 1100 nm, accuracy ± 0.5 nm; spectral bandwidth: 2 nm; light source: deuterium D2 / tungsten halogen lamp.

2 Experimental Procedures

Synthesis of 3,6-Diphenyl-1,4-dihydro-1,2,4,5-tetrazine (*d*TzPh$_2$, 2)

Synthesis modified from Fernández-Gómez.[3] PhCN (1.00 mL, 9.79 mmol, 1.0 eq.) was emulsified with $N_2H_4 \cdot H_2O$ (7.10 mL, 146 mmol, 15 eq.) and 3-mercaptopropionic acid (213 µL, 2.45 mmol, 0.25 eq.), and heated to 40 °C, resulting in a colourless solution. After stirring the solution at 40 °C for 66 hours it became an orange solid, which was cooled to rt, dissolved in CH_2Cl_2 (20 mL) and washed with an aqueous solution of NaOH (15 wt%, 3 × 10 mL). The organic layer was dried over Na_2SO_4, filtered, and evaporated to yield an orange solid (983 mg). Analysis by ^{1}H NMR spectroscopy revealed that the solid contained *d*TzPh$_2$ **2** and another compound assumed to be 3,5-diphenyl-4*H*-1,2,4-triazol-4-amine[3] in a molar ratio of 90:10 as the major components. The crude material was dissolved in EtOAc (50 mL) and was washed with an aqueous solution of HCl (1 M, 3 × 35 mL) and H_2O (35 mL), dried over $MgSO_4$, filtered, and evaporated to yield 3,6-diphenyl-1,4-dihydro-1,2,4,5-tetrazine (**2**) (848 mg, 3.59 mmol, 74%) as an orange solid in a mixture with minute amounts of EtOAc and silicon grease. ^{1}H NMR (500 MHz, CD_2Cl_2) δ [ppm]: 7.69 (m, 4H, *o*-C^{ar}**H**), 7.48 (m, 4H, *m*-C^{ar}**H**), 7.43 (m, 2H, *p*-C^{ar}**H**), 7.28 (br s, 2H, N**H**). ^{13}C{^{1}H} NMR (126 MHz {500 MHz}, CD_2Cl_2) δ [ppm]: 148.7 (**C**Ph), 131.0 (*p*-**C**^{ar}), 130.6 (**C**^{ar}Tz), 129.2 (*m*-**C**^{ar}), 126.3 (*o*-**C**^{ar}). HRMS (ESI, positive) *m*/*z*: calc. for $[C_{14}H_{12}N_4+H]^+$: 237.1135, exp: 237.1144. UV-Vis (CH_2Cl_2): λ [nm] (ε [$M^{-1}cm^{-1}$]) = 245 ($2.79 \cdot 10^4$), 291 ($4.25 \cdot 10^3$). Crystals, suitable for X-ray diffraction, were received by slow evaporation of a concentrated $CHCl_3$ solution at room temperature.

Synthesis of 3,6-Diphenyl-1,2,4,5-tetrazine ($TzPh_2$, 3)

Synthesis modified from Fernández-Gómez.[3] 3,6-Diphenyl-1,4-dihydro-1,2,4,5-tetrazine (**2**) (100 mg, 423 μmol, 1.0 eq.) was dissolved in MeOH (40 mL) and $Cu(OAc)_2 \cdot H_2O$ (8.44 mg, 42.3 μmol, 0.1 eq.) was added while bubbling air through the mixture with a cannula. The solution turned red immediately and changed to pink after several minutes. Concomitantly pink solid precipitated. After bubbling air through the mixture for two hours, the suspension was filtered over a Büchner funnel, washed with ice-cold MeOH, and the solid was dissolved in Et_2O before the solvents were evaporated. 3,6-Diphenyl-1,2,4,5-tetrazine (**3**) (69.0 mg, 295 μmol, 70%) was obtained as a pink solid. ^1H NMR (500 MHz, CD_2Cl_2) δ [ppm]: 8.65 (m, 4H, *o*-C^{ar}**H**), 7.70-7.60 (m, 6H, *m*-C^{ar}**H** and *p*-C^{ar}**H**). $^{13}C\{^1H\}$ NMR (126 MHz {500 MHz}, CD_2Cl_2) δ [ppm]: 164.5 (**C**Ph), 133.1 (*p*-$\mathbf{C}^{ar}$), 132.4 ($\mathbf{C}^{ar}$Tz), 129.7 (*m*-$\mathbf{C}^{ar}$), 128.3 (*o*-$\mathbf{C}^{ar}$). HRMS (ESI, positive) *m*/*z*: calc. for $[C_{14}H_{10}N_4+H]^+$: 235.0978, exp: 235.0987. UV-Vis (CH_2Cl_2): λ [nm] (ε [$M^{-1}cm^{-1}$]) = 297 ($2.85 \cdot 10^4$), 547 (465).

Synthesis of 2',5'-Di-*tert*-butyl-1,1':4',1''-terphenyl (*p*-Ter^{t}Bu$_2$)

Synthesis modified from Scheidt *et al.*[4] To a degassed solution of 2,2'-(2,5-di-*tert*-butyl-1,4-phenylene)bis(4,4,5,5-tetramethyl-1,3,2-dioxaborolane) (245 mg, 554 μmol, 1.5 eq.), K_2CO_3 (509 mg, 3.68 μmol, 10.0 eq.) and iodobenzene (41.1 μL, 368 μmol, 1.0 eq.) in H_2O (2 mL), EtOH (1 mL) and toluene (5 mL), $Pd(PPh_3)_4$ (85.2 mg, 73.7 μmol, 0.20 eq.) was added. The resulting yellow emulsion was stirred at 120 °C for 22.5 h, cooled to room temperature, and the layers were separated. The aqueous layer was extracted with EtOAc (2 × 20 mL), the combined organic layers washed with brine (20 mL), dried over $MgSO_4$, filtered and evaporated to obtain a brown solid (359 mg). Purification by column chromatography (l = 30 cm, d = 2 cm) using cyclohexane/EtOAc (10:1) to obtain a brown solid [R_f (cyclohexane/EtOAc, 10:1) = 0.55 – 0.30]. Purification by column chromatography (l = 35 cm, d = 2.5 cm) using cyclohexane/toluene (15:1 → 10:1) to obtain 2',5'-di-*tert*-butyl-1,1':4',1''-terphenyl (12.1 mg, 35.4 μmol, 10%) as a colourless solid [R_f (cyclohexane/toluene, 15:1) = 0.65]. ^1H NMR (500 MHz, CD_2Cl_2) δ [ppm]: 7.40 – 7.31 (m, 10H, $C^{ar,\ 2\text{-}6,2''\text{-}6''}$**H**), 7.11 (s, 2H, $C^{ar,\ 2',5'}$**H**), 1.14 (s, 18H, $C(C\mathbf{H}_3)_3$). $^{13}C\{^1H\}$ NMR (126 MHz {500 MHz}, CD_2Cl_2) δ [ppm]: 145.9, 144.4, and 141.1 ($\mathbf{C}^{ar,\ 1,1''}$, $\mathbf{C}^{ar,\ 1',4'}$, and $\mathbf{C}^{ar,\ 2',5'}$), 131.3 ($\mathbf{C}^{ar,\ 2',5'}$), 130.6 and 127.6 ($\mathbf{C}^{ar,\ 2,6,2',6'}$ and $\mathbf{C}^{ar,\ 3,5,3'',5''}$), 126.9 ($\mathbf{C}^{ar,\ 4,4''}$), 36.2 ($\mathbf{C}(CH_3)_3$), 32.8 ($C(\mathbf{C}H_3)_3$). ^1H NMR (600 MHz, toluene-d_8) δ [ppm]: 7.33 (m, 4H, $C^{ar,\ 2,6,2'',6''}$**H**), 7.15 (m, 4H, $C^{ar,\ 3,5,3'',5''}$**H**), 7.12 (m, 2H, $C^{ar,\ 4,4''}$**H**), 1.18 (s, 18H, $C(C\mathbf{H}_3)_3$). $^{13}C\{^1H\}$ NMR (126 MHz {500 MHz}, toluene-d_8) δ [ppm]: 146.6, 144.7, and 141.6 ($\mathbf{C}^{ar,\ 1,1''}$, $\mathbf{C}^{ar,\ 1',4'}$, and $\mathbf{C}^{ar,\ 2',5'}$), 131.8 ($\mathbf{C}^{ar,\ 2',5'}$), 130.9 and 128.0 ($\mathbf{C}^{ar,\ 2,6,2',6'}$ and $\mathbf{C}^{ar,\ 3,5,3'',5''}$), 127.2 ($\mathbf{C}^{ar,\ 4,4''}$), 36.5 ($\mathbf{C}(CH_3)_3$), 33.2 ($C(\mathbf{C}H_3)_3$). Crystals, suitable for X-ray diffraction, were received by slow evaporation of a concentrated CH_2Cl_2 solution at room temperature.

Synthesis of $TzPh_2$ 3 • BX_3 (X = Cl, Br)

To a pink solution of $TzPh_2$ **3** (1.0 eq.) in CD_2Cl_2 (~15 mL/mmol) BX_3 (1.0 eq., X = Cl: BCl_3 in CH_2Cl_2 (1 M), X = Br, BBr_3) was added, resulting in a reddish-orange solution. ^1H NMR spectroscopy confirmed successful adduct formation directly after addition. Removal of the solvents under reduced pressure and drying under high vacuum afforded $TzPh_2$ **3** • BX_3 as red oils (quant.), but this process was always accompanied by the formation of decomposition products, as indicated by ^1H NMR spectroscopic analyses (~5%, c.f. SI NMR spectrum 15).

TzPh$_2$ **3** • BCl$_3$: ^{1}H NMR (500 MHz, CD$_2$Cl$_2$) δ [ppm]: 8.54 (m, 4H, *o*-C^{ar}**H**), 7.82 (m, 2H, *p*-C^{ar}**H**), 7.72 (m, 4H, *m*-C^{ar}**H**). ^{13}C{^{1}H} NMR (126 MHz {500 MHz}, CD$_2$Cl$_2$) δ [ppm]: 159.6 (**C**Ph), 135.6 (*p*-**C**^{ar}), 131.0 (*o*-**C**^{ar}), 130.1 (*m*-**C**^{ar}), 129.3 (**C**^{ar}Tz). UV-Vis (CH$_2$Cl$_2$): λ [nm] (ε [M^{-1}cm^{-1}]) = 306 (2.01 • 10^4), 498 (691). Crystals of TzPh$_2$ **3** • BCl$_3$, suitable for X-ray diffraction, were received from a concentrated *ortho*-difluorobenzene solution at 7 °C after several days.

TzPh$_2$ **3** • BBr$_3$: ^{1}H NMR (500 MHz, CD$_2$Cl$_2$) δ [ppm]: 8.57 (m, 4H, *o*-C^{ar}**H**), 7.84 (m, 2H, *p*-C^{ar}**H**), 7.73 (m, 4H, *m*-C^{ar}**H**). ^{13}C{^{1}H} NMR (126 MHz {500 MHz}, CD$_2$Cl$_2$) δ [ppm]: 158.7 (**C**Ph), 135.9 (*p*-**C**^{ar}), 131.3 (*o*-**C**^{ar}), 130.1 (*m*-**C**^{ar}), 129.2 (**C**^{ar}Tz). UV-Vis (CH$_2$Cl$_2$): λ [nm] (ε [M^{-1}cm^{-1}]) = 230 (2.47 • 10^4), 256 (2.58 • 10^4), 506 (1.28 • 10^3). Crystals of TzPh$_2$ **3** • BBr$_3$ • 0.25 $C_6F_2H_4$, suitable for X-ray diffraction, were received from a saturated *ortho*-difluorobenzene solution at room temperature after one day. Crystals of *d*TzPh$_2$ **2** • 2 BBr$_3$, suitable for X-ray diffraction, were received from either a concentrated toluene solution (*d*TzPh$_2$ **2** • 2 BBr$_3$-α) or a concentrated dichloromethane/pentane (1:1) solution (*d*TzPh$_2$ **2** • 2 BBr$_3$-β) at room temperature after several days each.

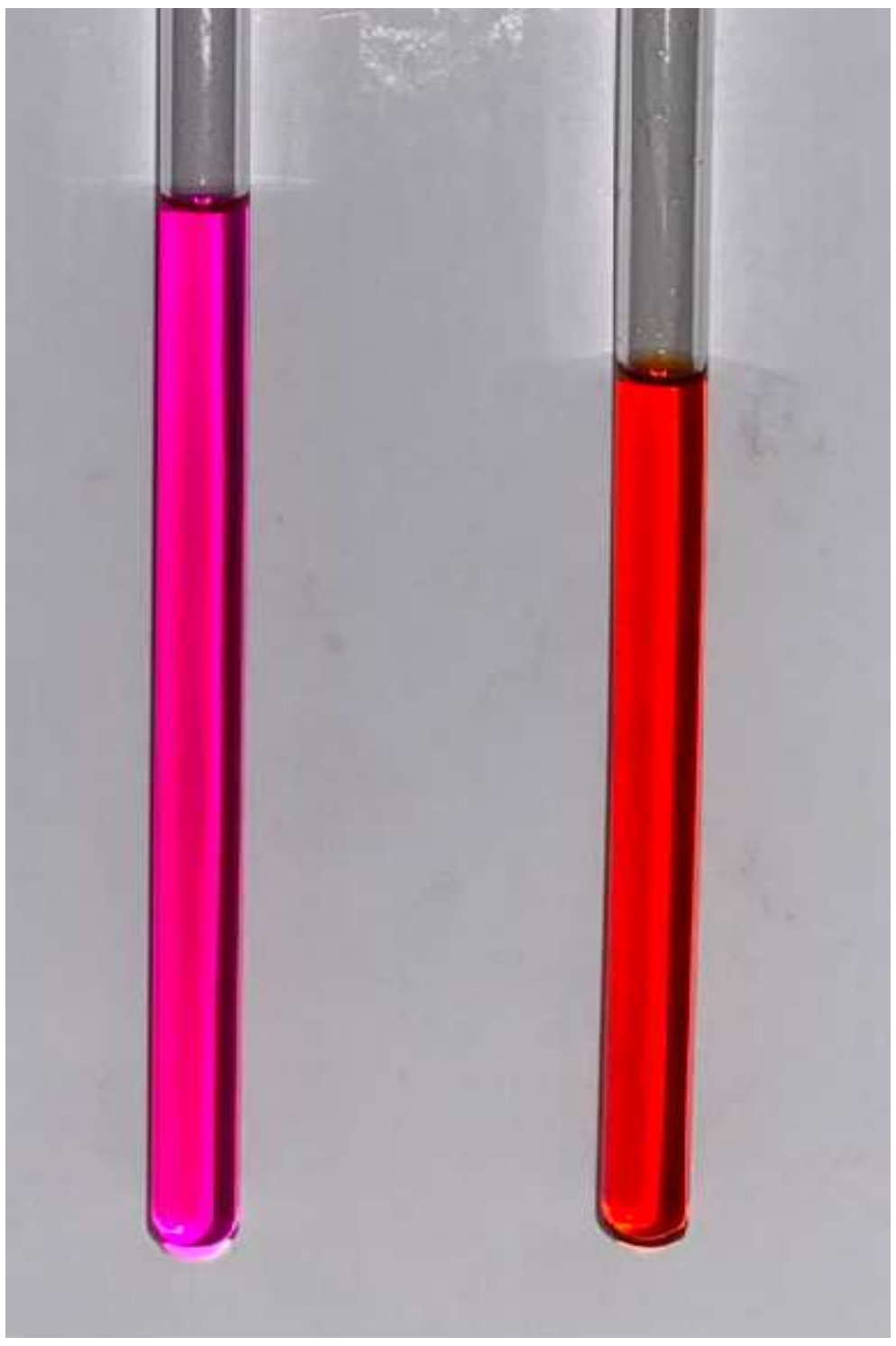

Figure S1 Photographs of TzPh$_2$ **3** in CD$_2$Cl$_2$ solution (left) and directly after addition of 1.0 eq. of BBr$_3$ (right).

3 ^{1}H DOSY NMR Spectroscopy

^{1}H diffusion NMR experiments have been performed on a BRUKER Avance *NEO* 600 FT NMR spectrometer, operating at a ^{1}H resonance frequency of 600.13 MHz at 293 K. The instrument was equipped with a 5 mm BBO Prodigy cryoprobe exhibiting a z-gradient coil delivering a maximum gradient strength g of 6.57 G mm^{-1} at 10 A. Diffusion data has been recorded using the dstebpgp3s pulse sequence delivered by the manufacturer. Diffusion coefficients D have been corrected according to the diffusion coefficient of H_2O (2.299 • 10^{-9} m^2 s^{-1} at 298 K) reported in the literature.[5] The corresponding proportional factor $D_{H2O,lit.}/D_{H2O,est.}$ of 0.98 was determined on a sample of acetone-d_6 equipped with a capillary containing H_2O. The temperature unit of the instrument was calibrated according to the manual of the manufacturer of the instrument using the temperature dependence of the proton chemical shift difference of methanol. To obtain stable temperature conditions, the sample was kept within the magnet for at least one hour at the respective temperature prior to data collection. ^{1}H-diffusion data were recorded with 32000 data points and a spectral width of 6580 Hz using a relaxation delay of 5 s. The diffusion delay time (big Delta, Δ) was set to 50 ms. The gradient duration time (little delta, δ/2) has been adjusted to values between 900 and 1100 µs. The gradient strength within the diffusion experiments was incremented linearly using 16 steps. The diffusion data has been analysed with the T_1/T_2 module which is part of the BRUKER TopSpin® software package. The standard deviation of the experimentally determined gradient strength-dependent signal intensities to the fitted decay function was ≤ 8.45 • 10^{-2}. DOSY plots presenting uncorrected diffusion constants in the F1 dimension have been prepared with MestreNova v14.3.2.

Solvodynamic radii r_s have been calculated by the Stokes-Einstein equation, adapted to small molecule conditions[6] as previously reported by Mitzel *et al.*[7],

$$D = \frac{k_B T}{c\pi\eta r_s} \quad (1)$$

$$r_s = \frac{k_B T}{c\pi\eta D} \quad (2)$$

with D = diffusion constant, k_B = 1.381 • 10^{-23} J K^{-1}[8] (Boltzmann constant), T = temperature, c = c-factor for small molecules, and η = dynamic viscosity. Since data for deuterated compounds are not available, the dynamic viscosity of the protonated solvent η(CH_2Cl_2 at 293 K) = 0.434 • 10^{-3} Pa s^{-1}[9] has been used instead. The c-factor for small molecules was calculated according to Chen *et al.*,[10]

$$c = \frac{6}{1 + 0.659\left(\frac{r_{solvent}}{r_{solute}}\right)^{2.234}} \quad (3)$$

with $r_{solvent}$ = van der Waals radius of the solvent and r_{solute} = van der Waals radius of the solute. Equations (1-2) typically contain the product of c and a shape factor for non-spherical molecules f_s (cf_s) as a term, but we have neglected the use of f_s because, firstly the substrates and adducts under investigation have different geometries, which would require calculations based on different assumptions, and secondly because the shape factor f_s is typically close to 1 anyway.[7]

Van der Waals radii r_{vdW} were calculated using the method reported by Abraham *et al.*,[11] firstly calculating the van der Waals volumes V_{vdW}:

$$V_{vdW}\ [\text{Å}^3] = \sum_i V_{vdW}^i - 5.92N_b - 14.7N_{ar} - 3.8N_{nar} \quad (4)$$

This empirically derived formula takes into account all atom contributions *i* of V_{vdW}, the number of bonds N_b (not differentiating between single and multiple bonds), number of aromatic rings N_{ar}, and number of non-aromatic rings N_{nar}. Van der Waals radii r_{vdW} are taken from Alvarez,[12]

Table S1 Selected atomic van der Waals radii (r_{vdW}) and volumes (V_{vdW}).[12]

	H	B	C	N	Cl	Br
r_{vdW} [Å]	1.20	1.91	1.77	1.66	1.82	1.86
V_{vdW} [Å^3]	7.24	29.2	23.2	19.2	25.3	27.0

and converted to van der Waals volumes V_{vdW} according to equations (5-6) (**Table S1**):

$$V = \frac{4}{3}\pi r^3 \quad (5)$$

$$r = \sqrt[3]{\frac{3V}{4\pi}} \quad (6)$$

Table S2 Calculated van der Waals radii (r_{vdW}) and volumes (V_{vdW}) and *c*-factor for small molecules with #X being the number of atoms of type X contained in the molecule, the number of bonds N_b, number of aromatic rings N_{ar}, and number of non-aromatic rings N_{nar}.

	#H	#B	#C	#N	#Cl	#Br	N_b	N_{ar}	N_{nar}	V_{vdW} [Å^3]	r_{vdW} [Å]	*c*
CH_2Cl_2	2	0	1	0	2	0	4	0	0	64.5	2.49	-
BCl_3	0	1	0	0	3	0	3	0	0	87.2	2.75	3.86
BBr_3	0	1	0	0	0	3	3	0	0	92.3	2.80	3.92
d$TzPh_2$ **2**	12	0	14	4	0	0	32	2	1	266	3.99	4.83
$TzPh_2$ **3**	10	0	14	4	0	0	30	3	0	253	3.92	4.79
p-Ter	14	0	18	0	0	0	34	3	0	274	4.03	4.85
p-Ter^tBu_2	30	0	26	0	0	0	58	3	0	434	4.70	5.14
$TzPh_2$ **3** • BBr_3	10	1	14	4	0	3	34	3	0	339	4.33	4.99
$TzPh_2$ **3** • 2 BBr_3	10	2	14	4	0	6	38	3	0	425	4.67	5.13
d$TzPh_2$ **2** • BBr_3	12	1	14	4	0	3	36	2	1	352	4.38	5.02
d$TzPh_2$ **2** • 2 BBr_3	12	2	14	4	0	6	40	2	1	439	4.71	5.14
$TzPh_2$ **3** • BCl_3	10	1	14	4	3	0	34	3	0	334	4.30	4.98
$TzPh_2$ **3** • 2 BCl_3	10	2	14	4	6	0	38	3	0	415	4.63	5.11

Table S3 Experimental diffusion constants D, corrected diffusion constants D_{corr} and calculated solvodynamic radii (r_s) and volumes (V_s) with their mean errors. a) The *p*-terphenyl scaffold seems to be an appropriate surrogate for the $TzPh_2$ scaffold judging from very similar volumes V_{vdW} (refer to **Table S2**) and V_s; b) calculated with c = 4.98 for $TzPh_2$ **3** • BCl_3; c) calculated with c = 5.11 for $TzPh_2$ **3** • 2 BCl_3; d) calculated with c = 4.99 for $TzPh_2$ **3** • BBr_3; e) calculated with c = 5.13 for $TzPh_2$ **3** • 2 BBr_3; f) calculated with c = 5.14 for *d*$TzPh_2$ **2** • 2 BBr_3; g) largest and smallest diffusion coefficients taken from the mixture (c.f. NMR spectrum ***22***); refer to **Table S2**.

	D [10^{-9} m^2 s^{-1}]	D_{corr} [10^{-9} m^2 s^{-1}]	r_s [Å]	V_s [Å^3]
d$TzPh_2$ **2**	1.39 ± 0.04	1.36 ± 0.04	4.52 ± 0.14	388 ± 12
$TzPh_2$ **3**	1.59 ± 0.05	1.56 ± 0.05	3.97 ± 0.12	262 ± 8
p-Ter [a)]	1.57 ± 0.05	1.54 ± 0.05	3.98 ± 0.12	263 ± 8
p-Ter^{t}Bu$_2$ [a)]	1.11 ± 0.03	1.08 ± 0.03	5.33 ± 0.16	634 ± 19
$TzPh_2$ **3** + 0.5 BCl_3 [b)]	1.48 ± 0.04	1.45 ± 0.04	4.09 ± 0.12	286 ± 9
$TzPh_2$ **3** + 1.0 BCl_3 [b)]	1.39 ± 0.04	1.36 ± 0.04	4.35 ± 0.13	344 ± 10
$TzPh_2$ **3** + 2.0 BCl_3 [c)]	1.22 ± 0.04	1.19 ± 0.04	4.82 ± 0.14	469 ± 14
$TzPh_2$ **3** + 2.5 BCl_3 [c)]	1.24 ± 0.04	1.21 ± 0.04	4.74 ± 0.14	446 ± 13
$TzPh_2$ **3** + 5.0 BCl_3 [c)]	1.19 ± 0.04	1.17 ± 0.04	4.93 ± 0.15	501 ± 15
$TzPh_2$ **3** + 0.5 BBr_3 [d)]	1.38 ± 0.04	1.35 ± 0.04	4.40 ± 0.13	358 ± 11
$TzPh_2$ **3** + 1.0 BBr_3 [d)]	1.20 ± 0.03	1.17 ± 0.03	5.07 ± 0.15	545 ± 16
$TzPh_2$ **3** + 2.0 BBr_3 [e)]	1.12 ± 0.03	1.09 ± 0.03	5.28 ± 0.16	618 ± 19
$TzPh_2$ **3** + 2.5 BBr_3 [e)]	1.13 ± 0.03	1.11 ± 0.03	5.23 ± 0.16	600 ± 18
$TzPh_2$ **3** + 5.0 BBr_3 [e)]	1.14 ± 0.03	1.12 ± 0.03	5.17 ± 0.16	580 ± 17
d$TzPh_2$ **2** + 2.5 BBr_3 [f), g)]	1.25 ± 0.04	1.22 ± 0.04	4.73 ± 0.14	442 ± 13
	0.915 ± 0.03	0.896 ± 0.03	6.44 ± 0.19	1118 ± 34
d$TzPh_2$ **2** + 5.0 BBr_3 [f), g)]	1.32 ± 0.04	1.30 ± 0.04	4.46 ± 0.13	373± 11
	1.02 ± 0.03	0.996 ± 0.03	5.81 ± 0.17	821± 25

Errors of diffusion constants D were approximated from the mean errors of the gradient-dependent intensity decay provided from DOSY measurements. By logarithmic plotting of normalized intensities $\ln(I/I_0)$ against g^2, the mean error from linear regression is averaged to be ~3%.

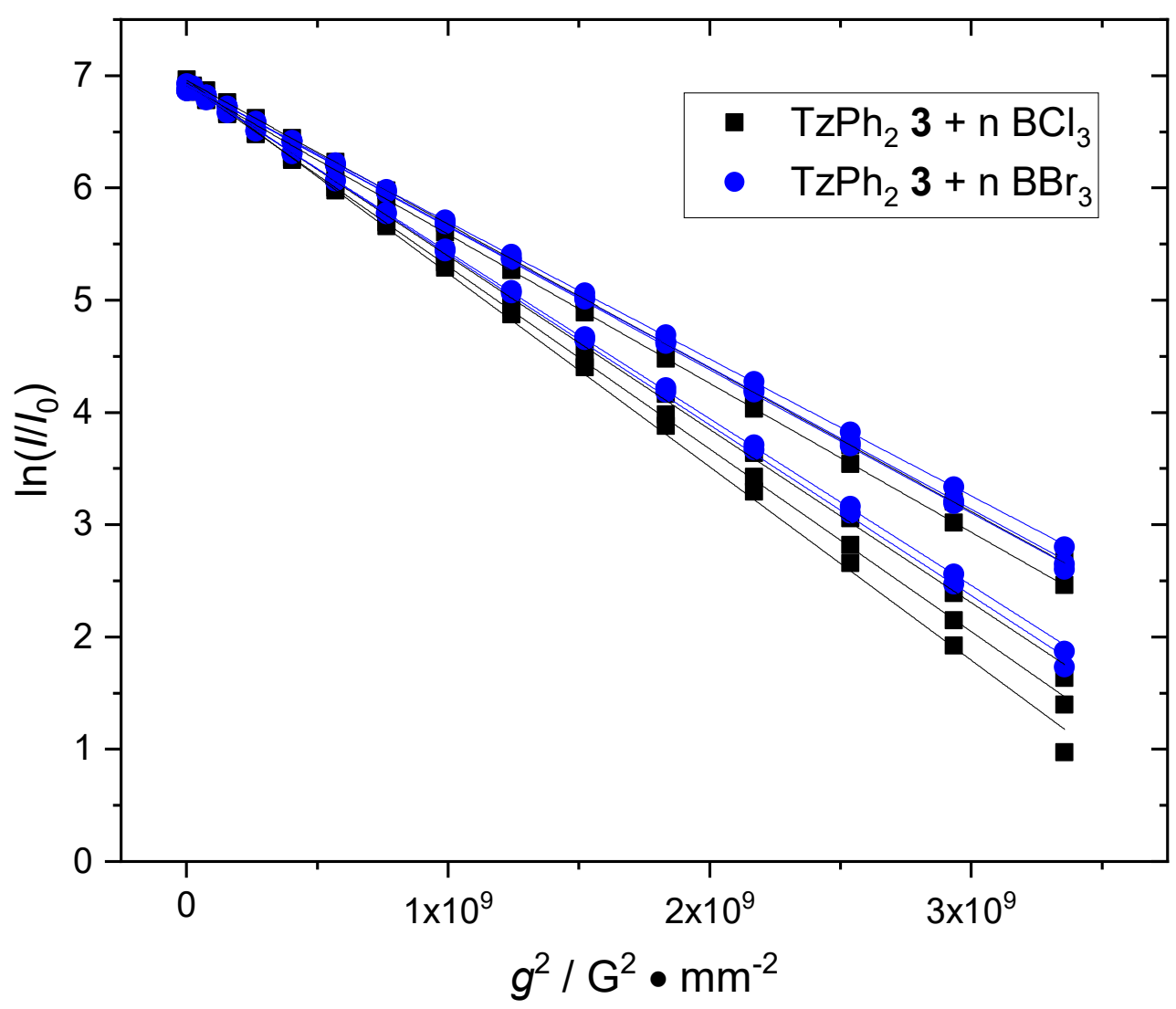

Figure S2 Plot of $\ln(I/I_0)$ against g^2 with linear regressions from ^{1}H DOSY measurements of $TzPh_2$ **3** • n BX_3 (X = Cl, Br, n = 0.5 – 5.0).

4 X-Ray Diffraction Data

Single crystals were examined on a Rigaku Supernova diffractometer using Cu Kα (λ = 1.54184 Å) radiation. The crystal was kept at 100.0(10) K during data collection.Using Olex2[2], the structures were solved with the SHELXT[13] structure solution program using Intrinsic Phasing. Refinement with the olex2.refine[14] refinement package using Gauss-Newton minimisation and NoSpherA2[15], an implementation of NOn-SPHERical Atom-form-factors in Olex2. Details of the X-ray investigations are given in Tables S4–S6. CCDC 2579984-2579989 contain the supplementary crystallographic data for this paper. These data can be obtained free of charge from The Cambridge Crystallographic Data Centre via www.ccdc.cam.ac.uk/conts/retrieving.html.

Table S4 Crystallographic data for compounds *d*TzPh$_2$ **2** • $CHCl_3$ and TzPh$_2$ **3** • BBr_3 • 0.25 $C_6F_2H_4$.

	***d*TzPh$_2$ 2 • $CHCl_3$**	**TzPh$_2$ 3 • BBr_3 • 0.25 $C_6F_2H_4$[a)]**
Empirical formula	$C_{15}H_{13}Cl_3N_4$	$C_{15.5}H_{11}BBr_3F_{0.5}N_4$
Formula weight	355.655	513.321
Temperature/K	100.00(10)	99.99(10)
Crystal system	monoclinic	monoclinic
Space group	$P2_1/c$	$I2/a$
a/Å	18.4834(4)	23.2175(5)
b/Å	5.4068(1)	11.9550(2)
c/Å	15.4742(4)	28.1512(6)
α/°	90	90
β/°	97.278(2)	116.629(3)
γ/°	90	90
Volume / Å^3	1533.97(6)	6985.0(3)
Z	4	16
ρ_{calc} g/cm^3	1.540	1.953
μ / mm^{-1}	5.416	8.661
F(000)	734.1	3920.5
Crystal size / mm^3	0.53 × 0.09 × 0.04	0.07 × 0.06 × 0.03
Radiation / Å	Cu K_α (λ = 1.54184)	Cu K_α (λ = 1.54184)
2Θ range for data collection / °	11.54 to 153.46	7.02 to 151.68
Index ranges	$-23 \le h \le 23$, $-6 \le k \le 6$, $-19 \le l \le 19$	$-17 \le h \le 29$, $-15 \le k \le 13$, $-34 \le l \le 28$
Reflections collected	24614	19411
Independent reflections	3205 [R_{int} = 0.0685, R_{sigma} = 0.0282]	7181 [R_{int} = 0.0454, R_{sigma} = 0.0523]
Reflections with $I \ge 2u(I)$	2969	5934
Data / restraints / parameters	3205/0/251	7181/54/469
Goodness-of-fit on F^2	1.059	1.024
Final R indexes [$I \ge 2u(I)$]	R1 = 0.0297, wR2 = 0.0795	R_1 = 0.0342, wR_2 = 0.0775
Final R indexes [all data]	R1 = 0.0318, wR2 = 0.0821	R_1 = 0.0463, wR_2 = 0.0828
Largest diff. peak/hole / e·Å^{-3}	0.32/-0.38	0.96/−1.02
CCDC number	2579984	2579985

a) Disorder of one *ortho*-difluorobenzene molecule (C29-34; F1/2) at an inversion center.

Table S5 Crystallographic data for compounds *d*TzPh$_2$ **2** • 2 BBr_3-α and *d*TzPh$_2$ **2** • 2 BBr_3-β.

	***d*TzPh$_2$ 2 • 2 BBr$_3$-α**	***d*TzPh$_2$ 2 • 2 BBr$_3$-β**[a)]
Empirical formula	$C_{14}H_{12}B_2Br_6N_4$	$C_{14}H_{12}B_2Br_6N_4$
Formula weight	737.348	737.36
Temperature/K	100.00(10)	100.00(10)
Crystal system	monoclinic	monoclinic
Space group	$P2_1/c$	$P2_1/n$
a/Å	21.65944(13)	15.80964(6)
b/Å	6.96243(5)	18.00237(7)
c/Å	14.59005(11)	22.60640(9)
α/°	90	90
β/°	100.4360(6)	96.4680(4)
γ/°	90	90
Volume / Å^3	2163.82(3)	6393.07(4)
Z	4	12
ρ_{calc} g/cm^3	2.263	2.298
μ / mm^{-1}	13.465	13.672
F(000)	1362.1	4128.0
Crystal size / mm^3	0.28 × 0.20 × 0.19	0.28 × 0.23 × 0.13
Radiation / Å	Cu K_α (λ = 1.54184)	Cu K_α (λ = 1.54184)
2Θ range for data collection / °	12.28 to 152	6.292 to 153.118
Index ranges	$-27 \le h \le 27$, $-8 \le k \le 8$, $-18 \le l \le 18$	$-19 \le h \le 19$, $-22 \le k \le 22$, $-28 \le l \le 28$
Reflections collected	123301	231802
Independent reflections	4520 [R_{int} = 0.0576, R_{sigma} = 0.0120]	13411 [R_{int} = 0.0471, R_{sigma} = 0.0127]
Reflections with $I \ge 2u(I)$	4515	13373
Data / restraints / parameters	4520/0/284	13411/0/847
Goodness-of-fit on F^2	1.024	1.000
Final R indexes [$I \ge 2u(I)$]	R_1 = 0.0298, wR_2 = 0.0743	R1 = 0.0260, wR2 = 0.0624
Final R indexes [all data]	R_1 = 0.0298, wR_2 = 0.0743	R1 = 0.0261, wR2 = 0.0625
Largest diff. peak/hole / e·Å^{-3}	1.07/−0.58	2.02/-1.05
CCDC number	2579986	2579987

a) All hydrogen atoms were refined isotropically.

Table S6 Crystallographic data for compounds $TzPh_2$ **3** • BCl_3 and *p*-Ter^{t}Bu$_2$.

	**$TzPh_2$ 3 • BCl_3**[a)]	***p*-Ter^{t}Bu$_2$**
Empirical formula	$C_{14}H_{10}BCl_3N_4$	$C_{26}H_{30}$
Formula weight	351.444	342.528
Temperature/K	100.00(10)	100.00(10)
Crystal system	tetragonal	triclinic
Space group	$I4_1/a$	$P\overline{1}$
a/Å	32.4532(9)	5.8647(3)
b/Å	32.4532(9)	9.3315(5)
c/Å	11.5254(7)	10.0169(5)
α/°	90	106.850(4)
β/°	90	106.002(4)
γ/°	90	98.126(4)
Volume / Å^3	12138.6(9)	489.52(5)
Z	32	1
ρ_{calc} g/cm^3	1.538	1.162
μ / mm^{-1}	5.462	0.482
F(000)	5745.8	186.5
Crystal size / mm^3	0.24 × 0.05 × 0.04	0.29 × 0.19 × 0.15
Radiation / Å	Cu K_α (λ = 1.54184)	Cu K_α (λ = 1.54184)
2Θ range for data collection / °	5.44 to 152.7	11.44 to 151.76
Index ranges	$-40 \le h \le 40$, $-40 \le k \le 40$, $-14 \le l \le 13$	$-7 \le h \le 7$, $-11 \le k \le 11$, $-12 \le l \le 12$
Reflections collected	48847	7431
Independent reflections	6340 [R_{int} = 0.0896, R_{sigma} = 0.0484]	2018 [R_{int} = 0.0244, R_{sigma} = 0.0183]
Reflections with $I \ge 2u(I)$	4879	1871
Data / restraints / parameters	6340/0/477	2018/0/121
Goodness-of-fit on F^2	1.028	1.087
Final R indexes [$I \ge 2u(I)$]	R_1 = 0.0296, wR_2 = 0.0531	R_1 = 0.0249, wR_2 = 0.0634
Final R indexes [all data]	R_1 = 0.0497, wR_2 = 0.0588	R_1 = 0.0269, wR_2 = 0.0649
Largest diff. peak/hole / e·Å^{-3}	0.44/−0.37	0.13/−0.20
CCDC number	2579988	2579989

a) Crystals were picked from the mother liquor, selected and mounted under a stream of dry N_2 and Santovac cryo oil each cooled to 240 K.

Molecular Volumes extracted from SC-XRD measurements (V_{XRD})

Volumes extracted from the solid-state structure determined by XRD measurements V_{XRD} have been calculated from the unit cells' volumes, optionally subtracting the contribution of co-crystallised solvent molecules using data from literature. The volumes V_{XRD} are reported with their standard deviations.

Table S7 Molecular Volumes V_{XRD} with standard deviation. * The contribution of co-crystallised 1.0 $CHCl_3$ was subtracted using data from the SC-XRD measurement of sole $CHCl_3$.[17] ** The contribution of co-crystallised 0.25 *ortho*-difluorobenzene was subtracted using data from the SC-XRD measurement of sole *ortho*-difluorobenzene.[18]

	V_{XRD} [Å^3]
*d*TzPh$_2$ **2**	283.5 ± 0.3*
TzPh$_2$ **3**[19]	272.6 ± 0.3
p-Ter[20]	298.4 ± 0.3
p-Ter^{t}Bu$_2$	489.5 ± 0.5
BBr_3[21]	122.6 ± 0.1
TzPh$_2$ **3** • BCl_3	379.3 ± 0.1
TzPh$_2$ **3** • BBr_3	404.8 ± 0.5**
*d*TzPh$_2$ **2** • 2 BBr_3-α	541.0 ± 0.5
*d*TzPh$_2$ **2** • 2 BBr_3-β	532.8 ± 0.9

Crystal Structure of *d*TzPh$_2$ 2 • $CHCl_3$

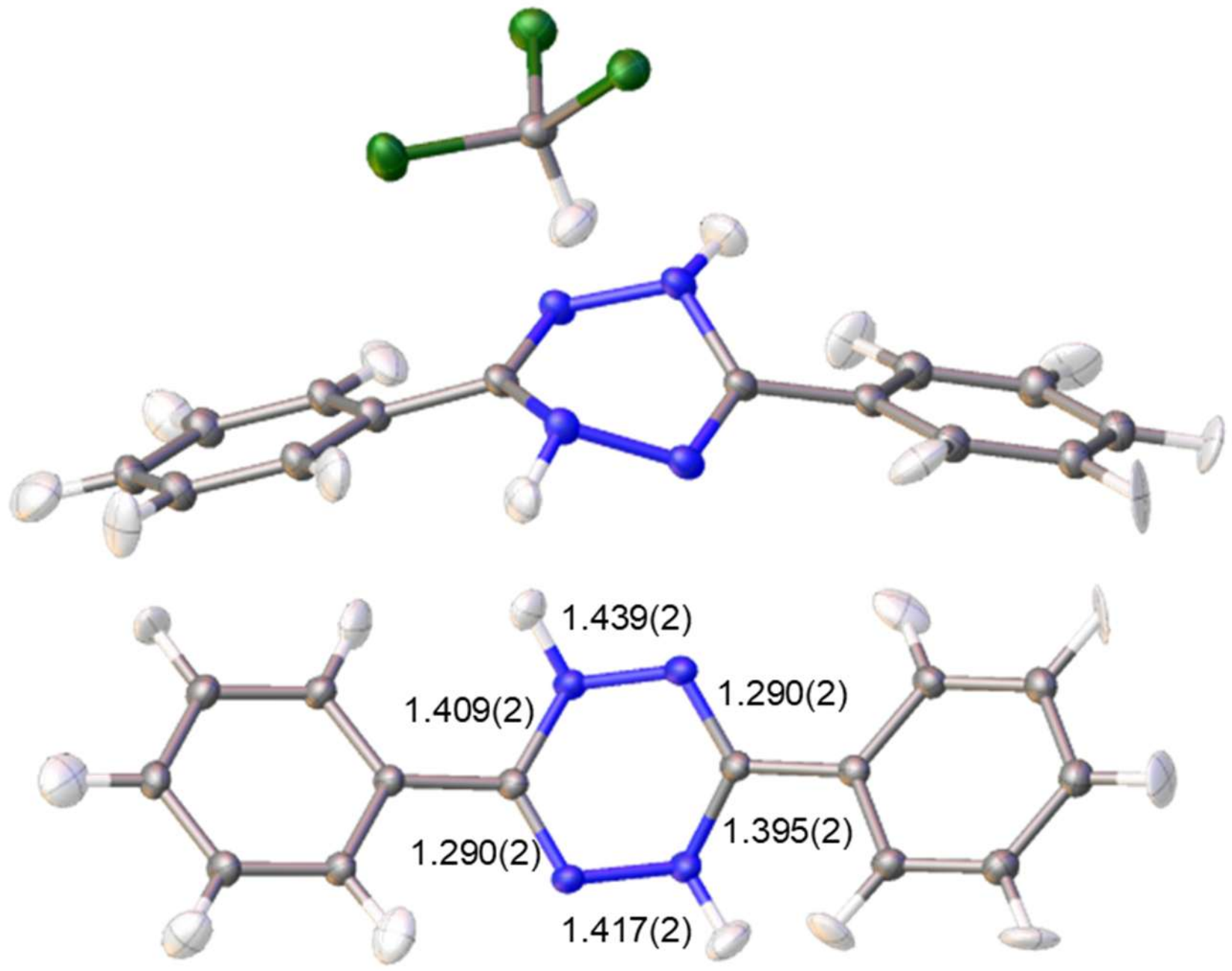

Figure S3: Molecular structure of *d*TzPh$_2$ **2** • $CHCl_3$ as determined by SC-XRD measurements. Thermal ellipsoids depicted at 50% probability. Top: structure of *d*TzPh$_2$ • $CHCl_3$ in the asymmetric unit; bottom: excerpt of the *d*TzPh$_2$ unit with selected bond lengths (in Å).

Crystal Structure of *p*-Ter^{t}Bu$_2$

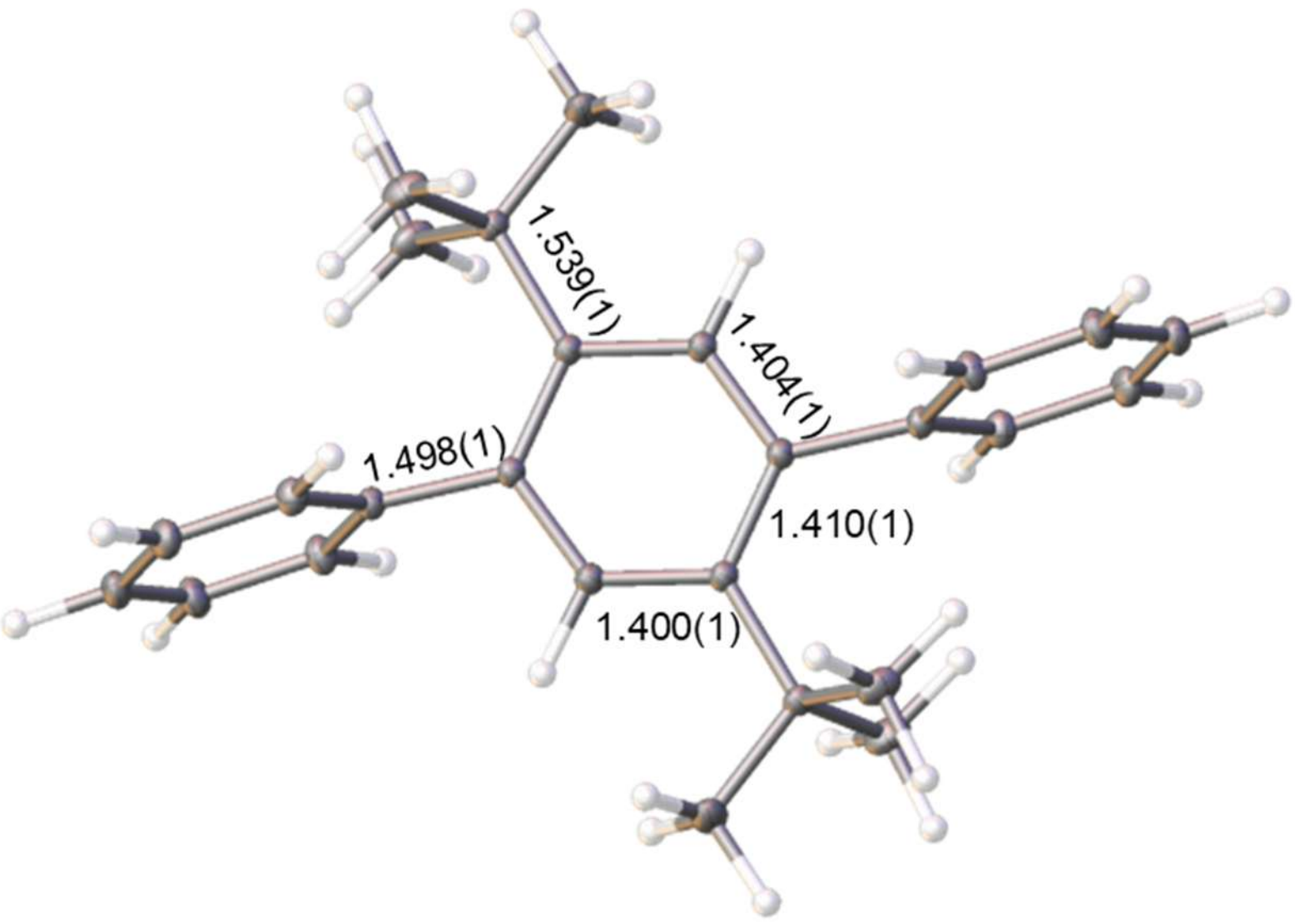

Figure S4: Molecular structure of *p*-Ter^{t}Bu$_2$ as determined by SC-XRD measurements. Thermal ellipsoids depicted at 50% probability. Selected bond lengths (in Å, Z' = 0.5, Symmetry code: 2−*X*, 1−*Y*, 1−*Z*).

Crystal Structure of *d*TzPh$_2$ 2 • 2 BBr$_3$-β

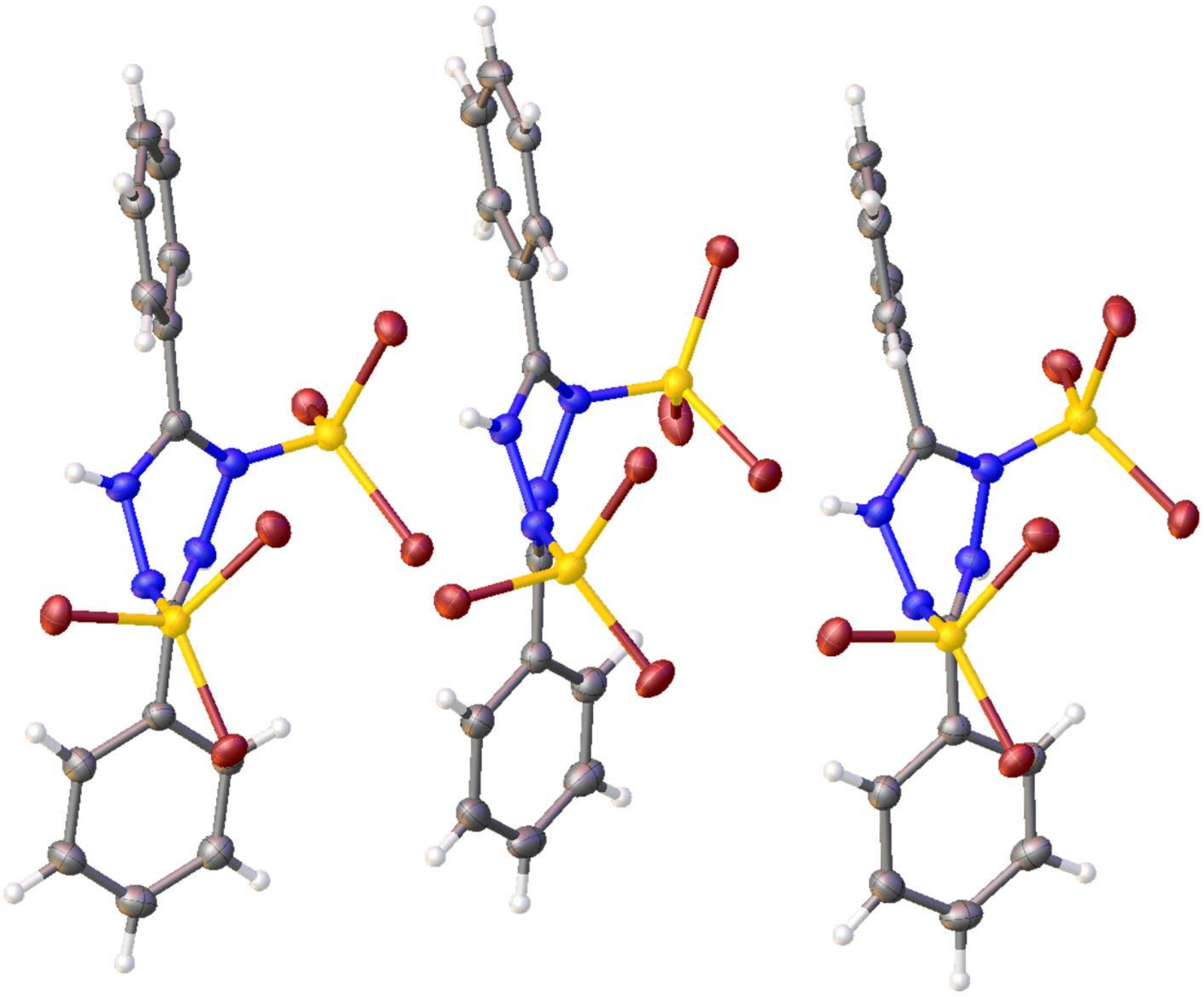

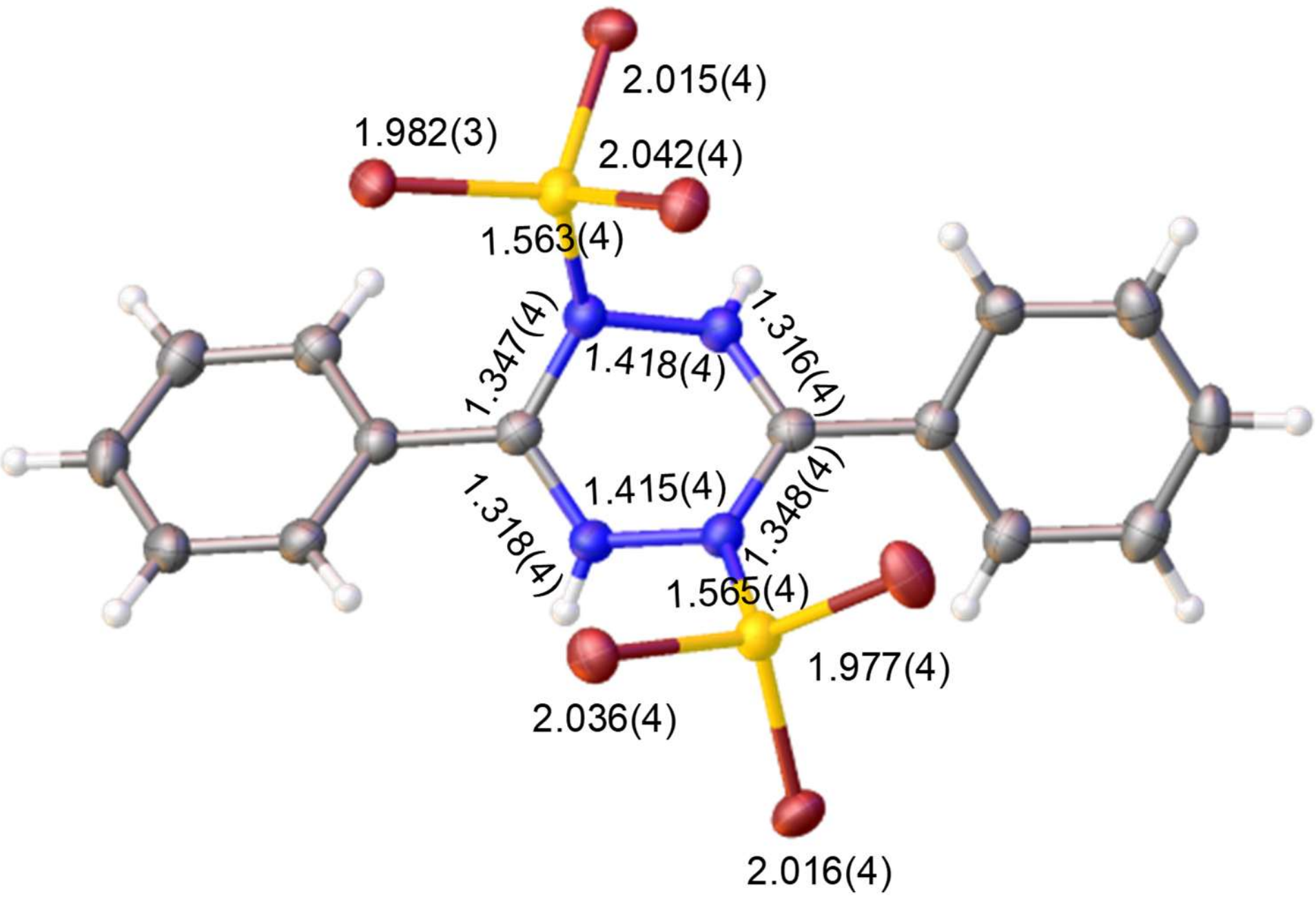

Figure S5: Molecular structure of *d*TzPh$_2$ **2** • 2 BBr$_3$-β as determined by SC-XRD measurements. Thermal ellipsoids depicted at 50% probability. Top: structure of *d*TzPh$_2$ **2** • 2 BBr$_3$-β in the asymmetric unit; bottom: excerpt of one representative *d*TzPh$_2$ • 2 BBr$_3$ unit with selected bond lengths (in Å, lengths and angles match those described for the other molecules in the asymmetric unit within a 3σ margin of error).

5 UV-Vis Spectroscopy

Samples for UV-Vis spectroscopic measurements were prepared in dichloromethane at 100 µM under an inert argon atmosphere.

Table S8 Absorption maxima λ_{max} and molar attenuation coefficients ε of selected compounds and compound mixtures.

	λ_{max} [nm]	ε [M^{-1} cm^{-1}]
*d*TzPh₂ **2**	245	$2.79 \cdot 10^4$
	291	$4.25 \cdot 10^3$
TzPh₂ **3**	297	$2.85 \cdot 10^4$
	547	465
*d*TzPh₂ **2** + 2.5 BBr_3	230	$2.47 \cdot 10^4$
	256	$2.58 \cdot 10^4$
	288	$1.05 \cdot 10^4$
TzPh₂ **3** + 2.5 BCl_3	306	$2.01 \cdot 10^4$
	498	691
TzPh₂ **3** + 2.5 BBr_3	230	$2.47 \cdot 10^4$
	256	$2.58 \cdot 10^4$
	506	$1.28 \cdot 10^3$

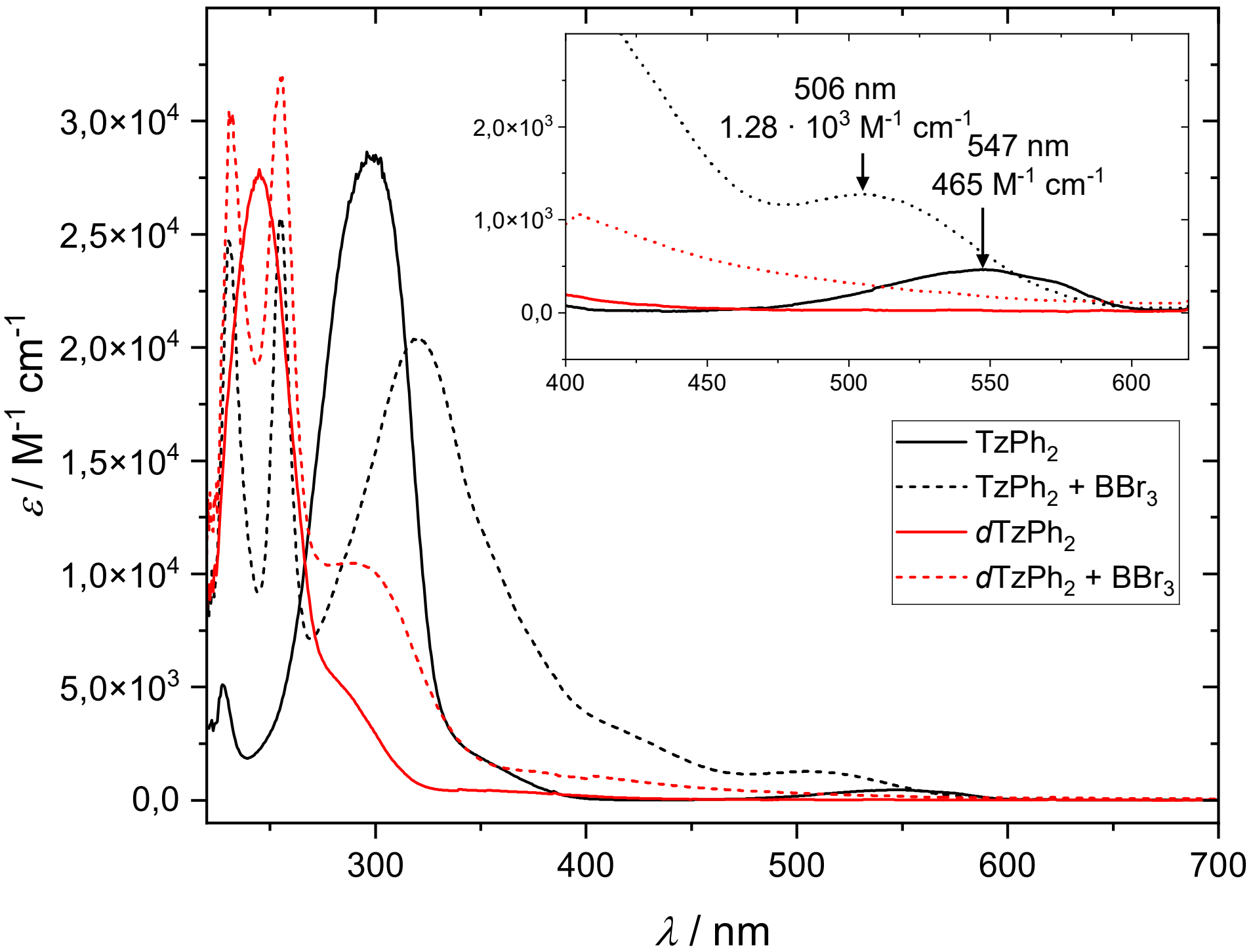

Figure S6 Comparison of UV-Vis spectra of TzPh₂ **3** and the adduct TzPh₂ **3** • BBr_3 with *d*TzPh₂ **2** and *d*TzPh₂ **2** mixed with BBr_3 (2.5 eq.) in dichloromethane.

6 NMR Spectra

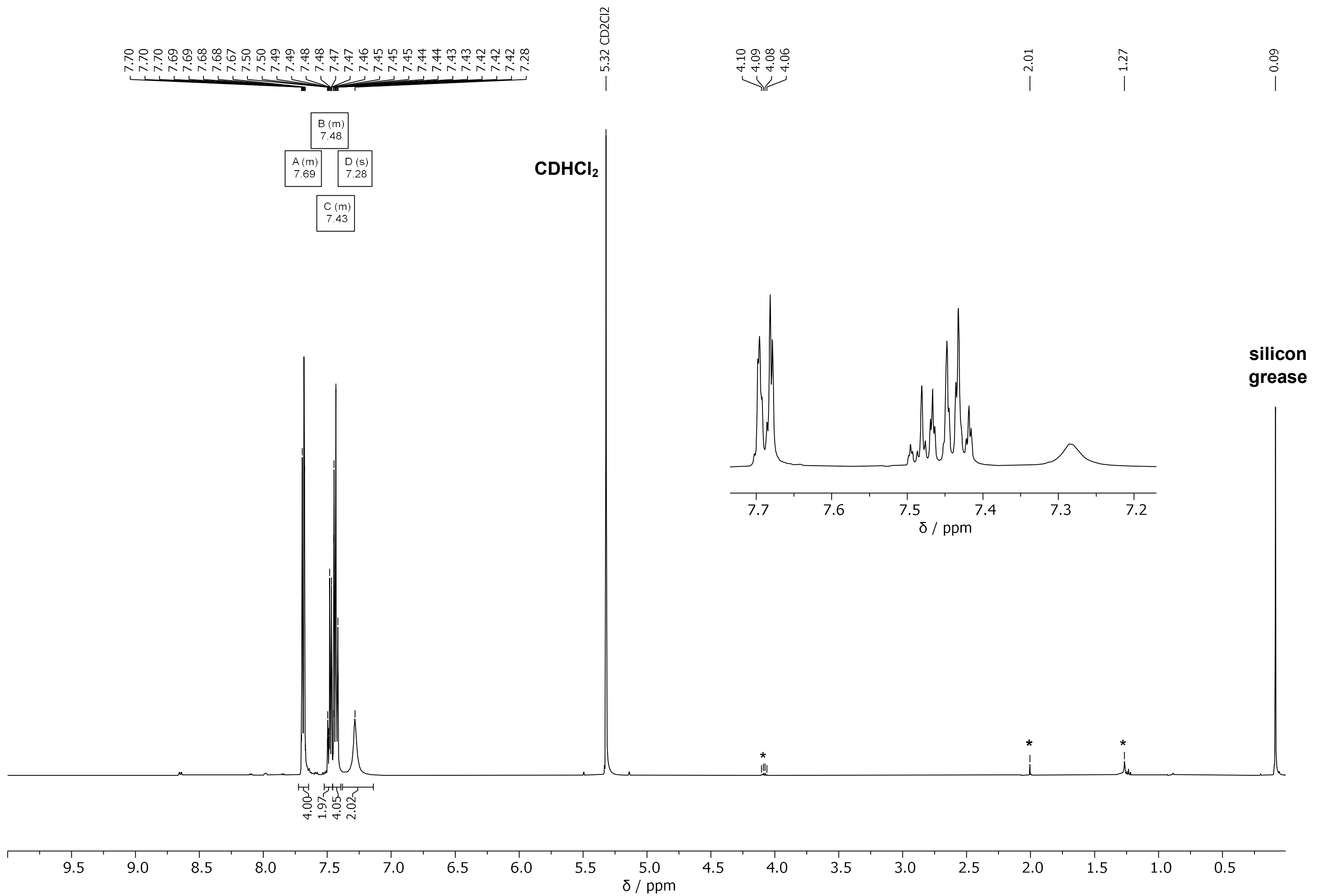

NMR spectrum *1*: ^{1}H NMR spectrum (500 MHz, dichloromethane-d_2) of *d*TzPh_2 **2** in mixture with traces of EtOAc (*).

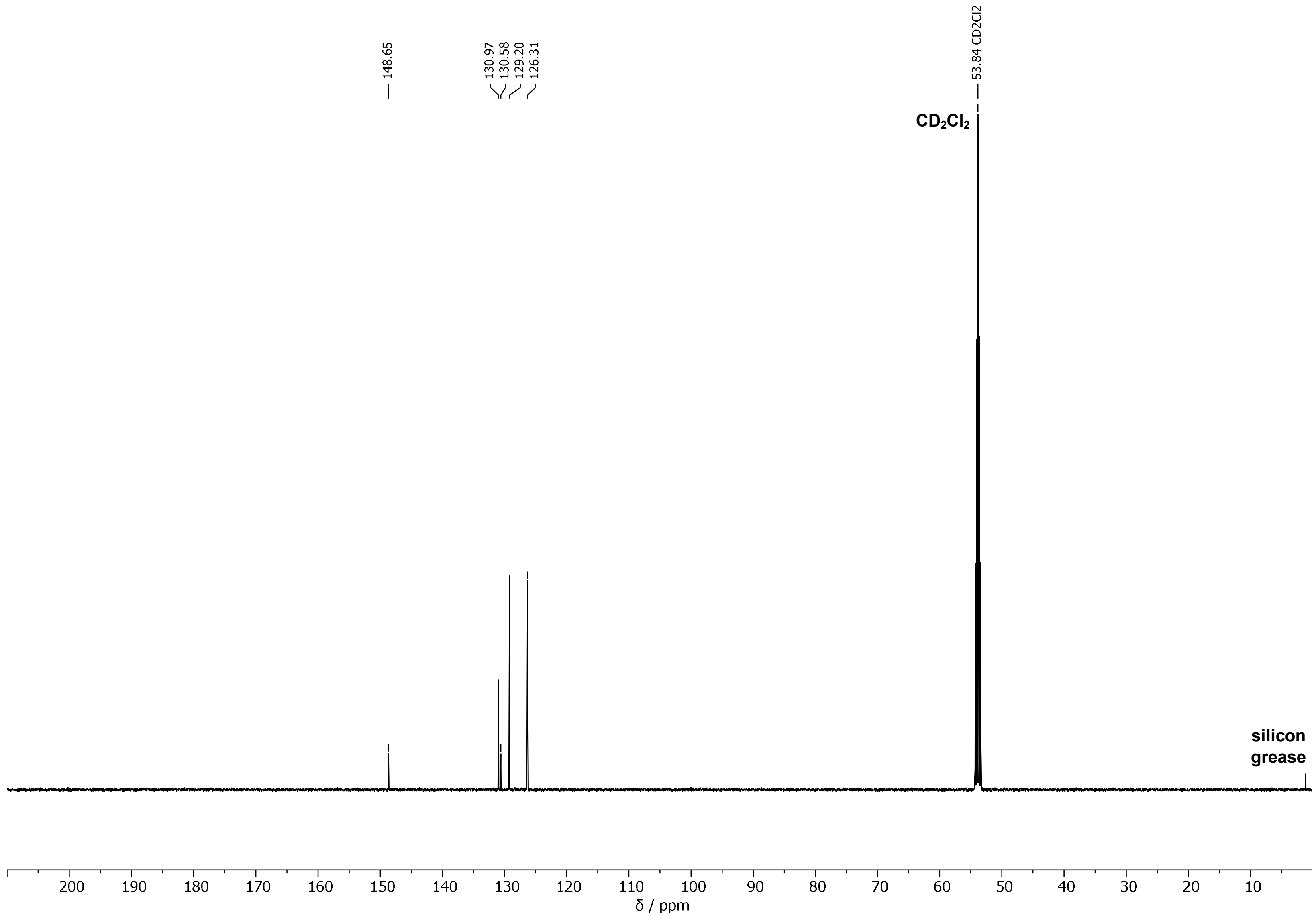

NMR spectrum *2***:** $^{13}C\{^{1}H\}$ NMR spectrum (126 {500} MHz, dichloromethane-d_2) of *d*TzPh$_2$ **2**.

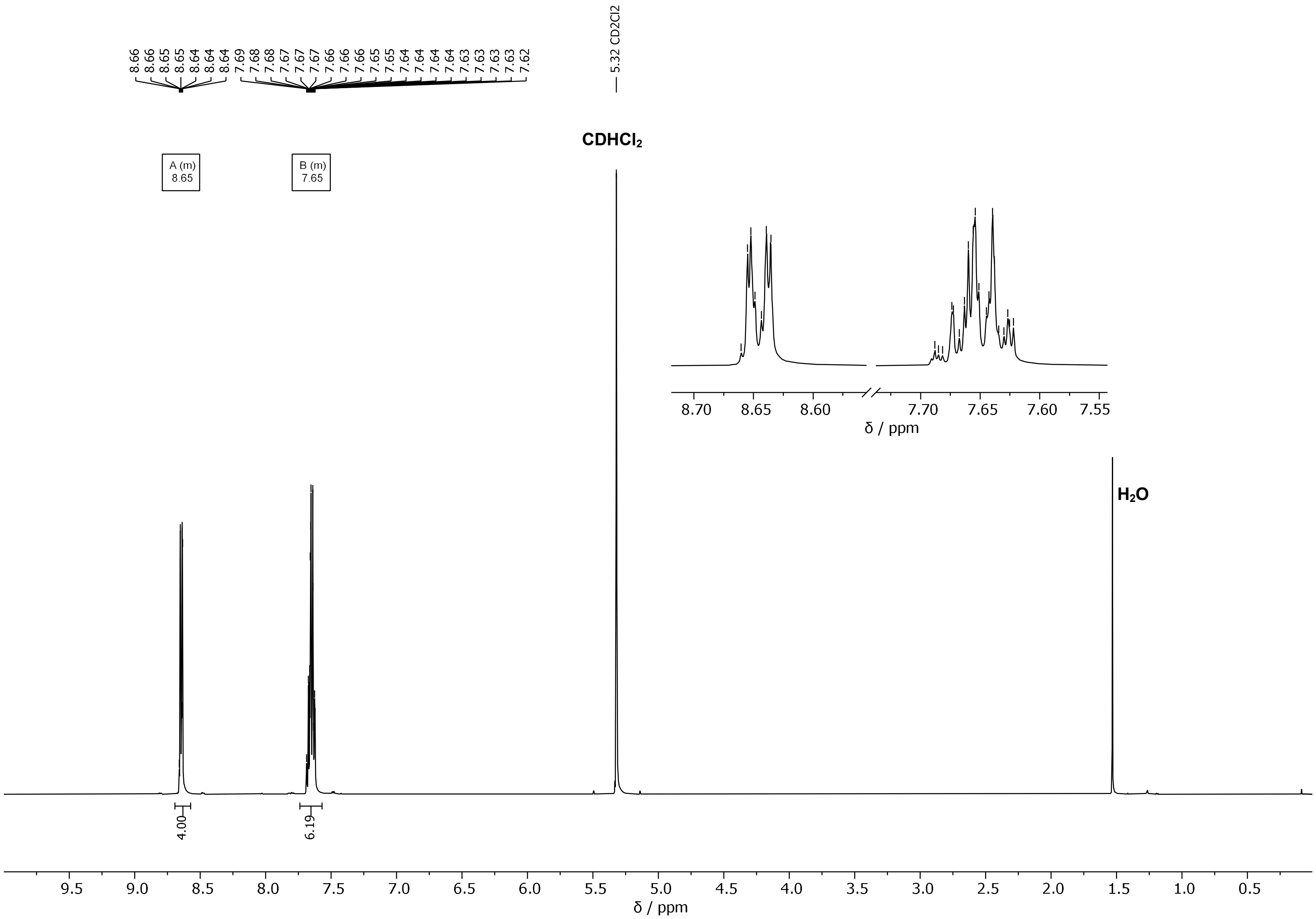

NMR spectrum *3*: ^{1}H NMR spectrum (500 MHz, dichloromethane-d_2) of $TzPh_2$ **3**.

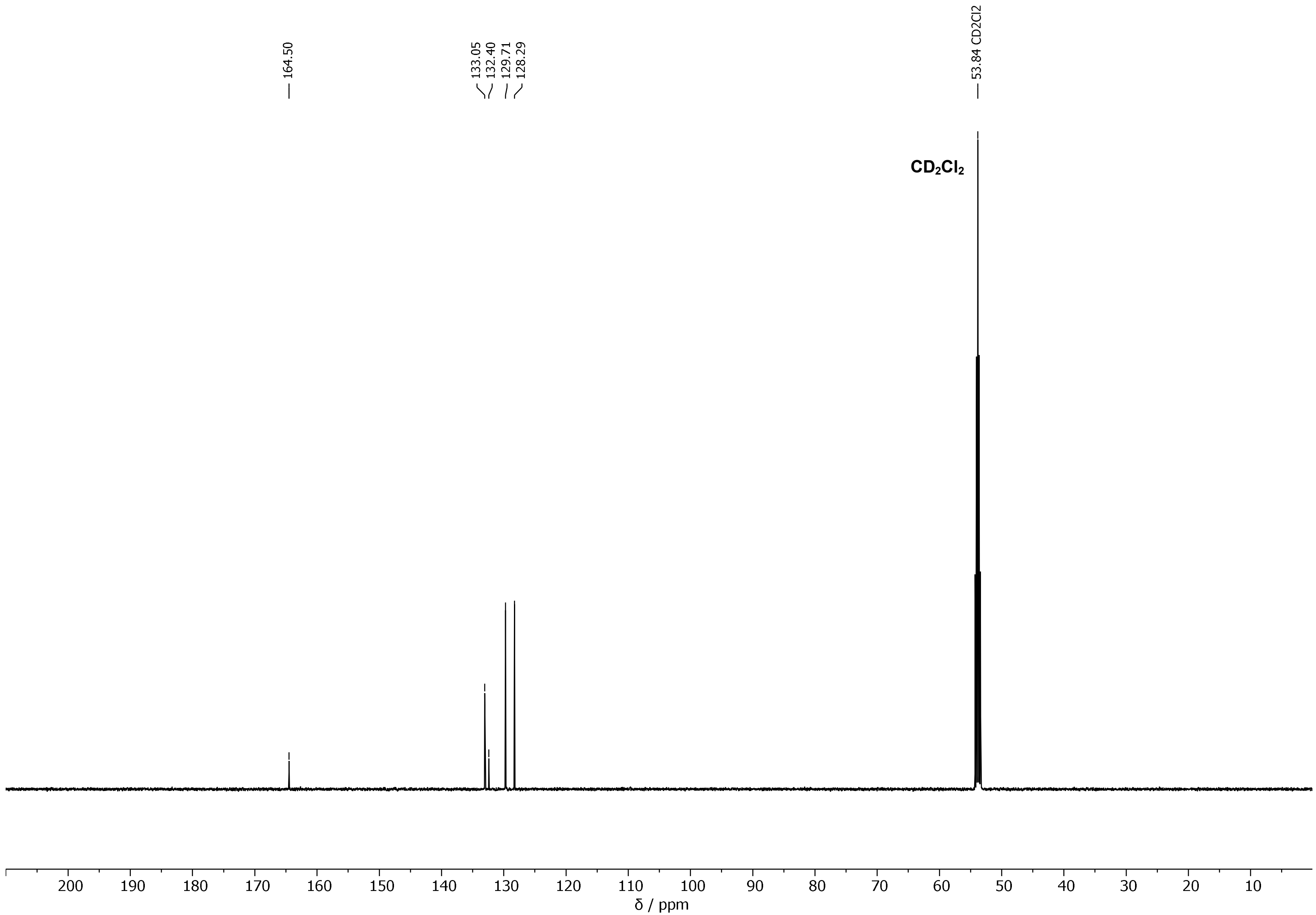

NMR spectrum *4*: $^{13}C\{^{1}H\}$ NMR spectrum (126 {500} MHz, dichloromethane-d_2) of $TzPh_2$ **3**.

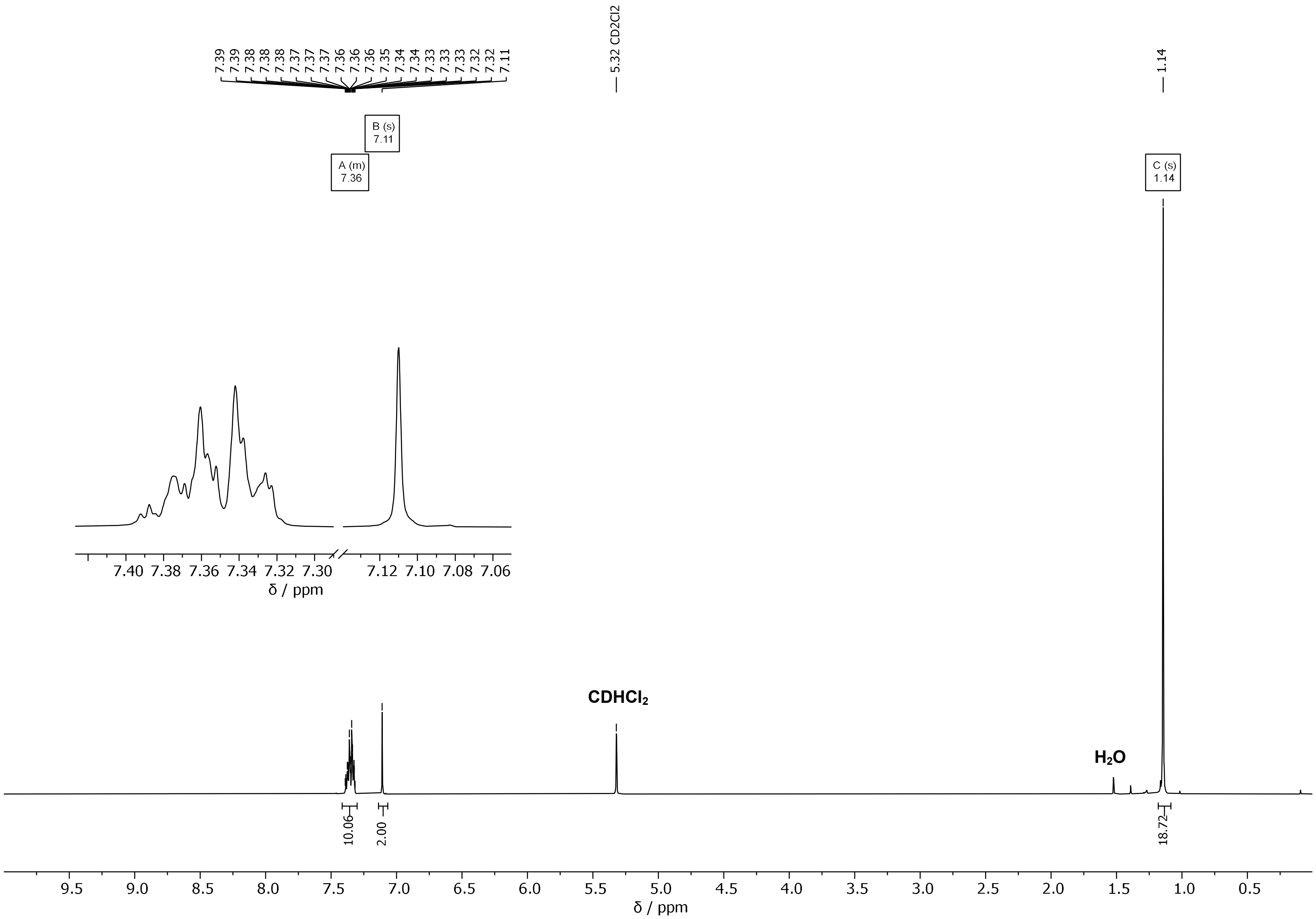

NMR spectrum *5*: ^{1}H NMR spectrum (500 MHz, dichloromethane-d_2) of 2',5'-di-*tert*-butyl-1,1':4',1''-terphenyl.

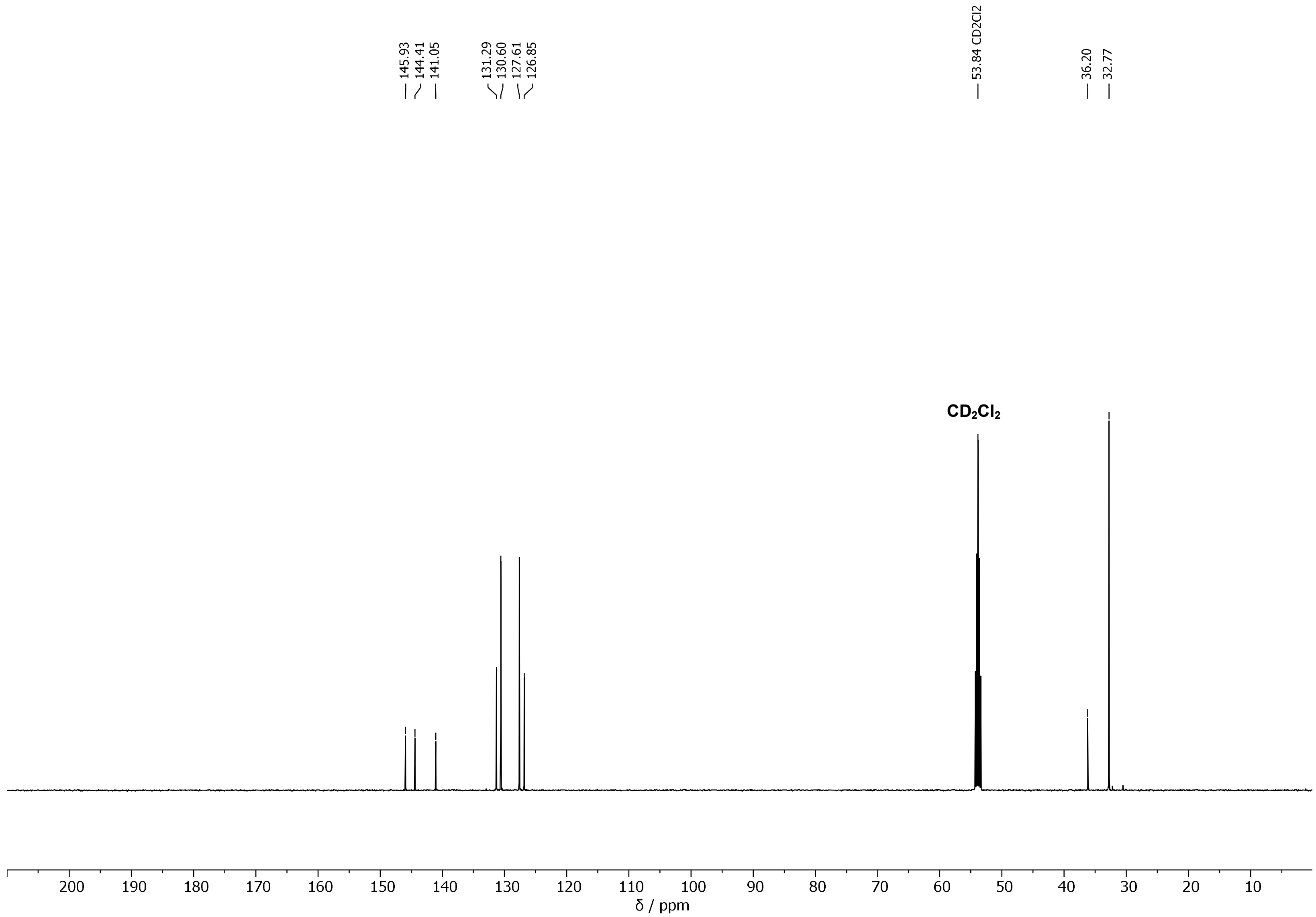

NMR spectrum *6*: $^{13}C\{^{1}H\}$ NMR spectrum (126 {500} MHz, dichloromethane-d_2) of 2',5'-di-*tert*-butyl-1,1':4',1''-terphenyl.

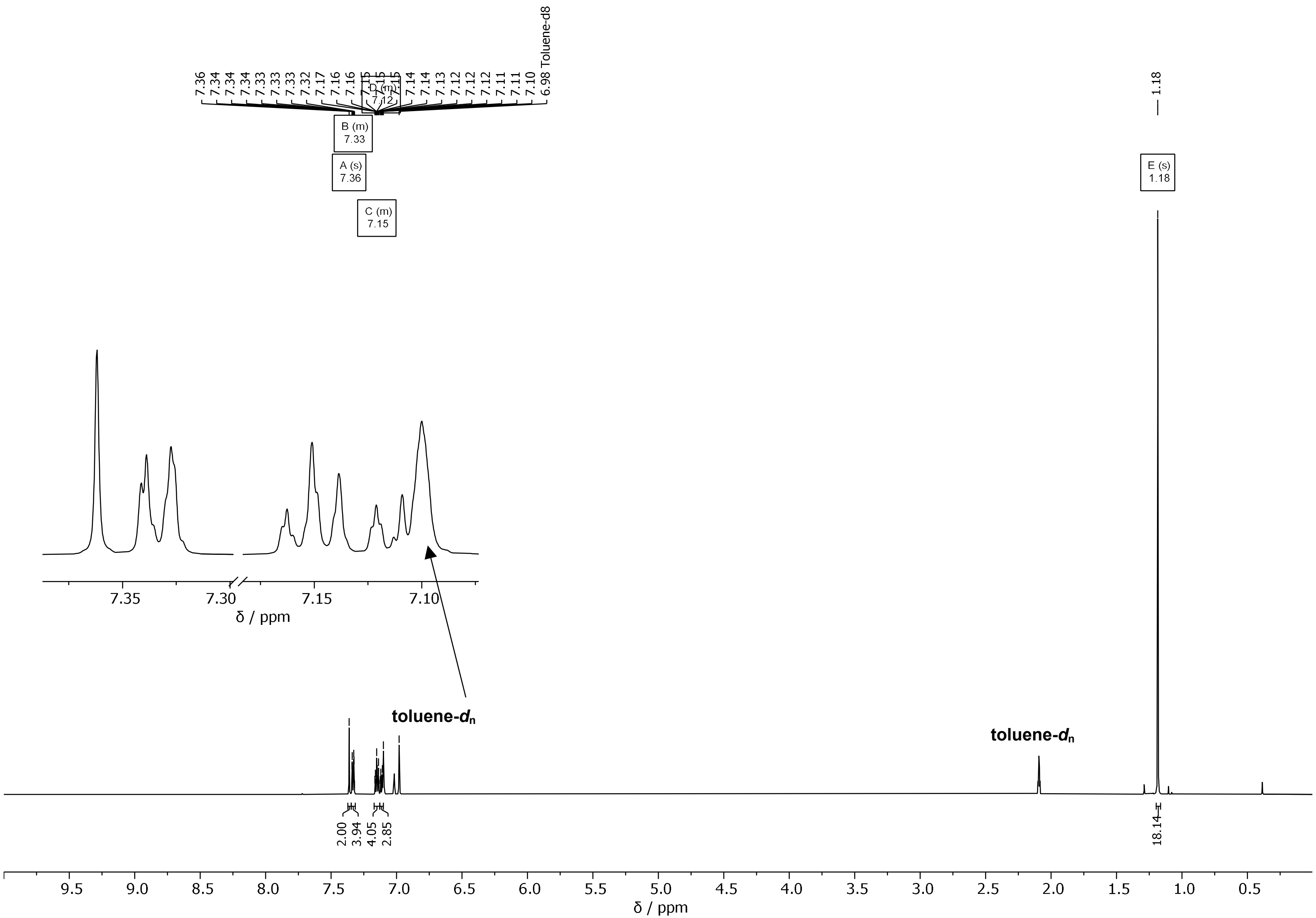

NMR spectrum *7***:** ^{1}H NMR spectrum (600 MHz, toluene-d_8) of 2',5'-di-*tert*-butyl-1,1':4',1''-terphenyl.

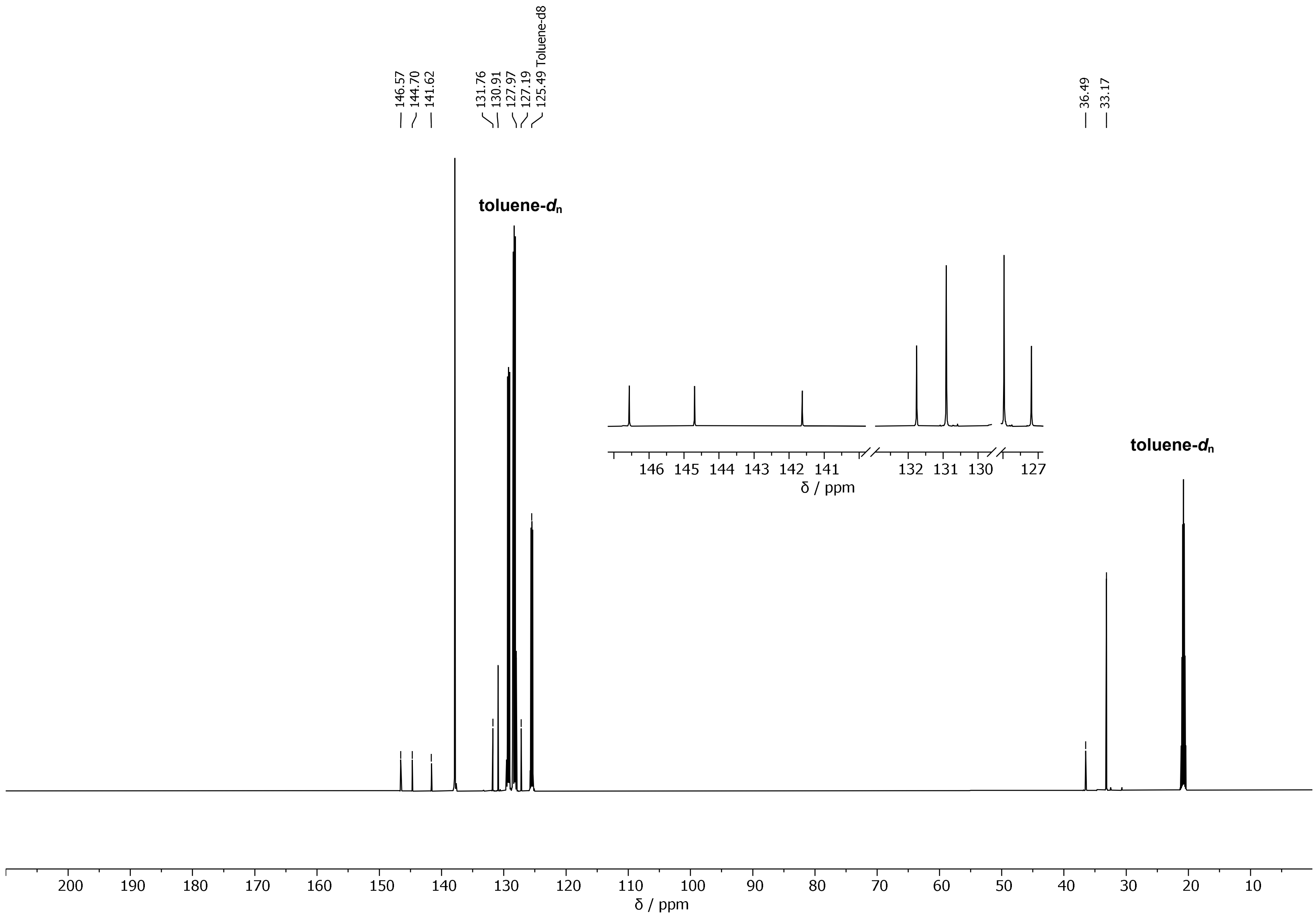

NMR spectrum *8*: $^{13}C\{^1H\}$ NMR spectrum (151 {600} MHz, toluene-d_8) of 2',5'-di-*tert*-butyl-1,1':4',1''-terphenyl.

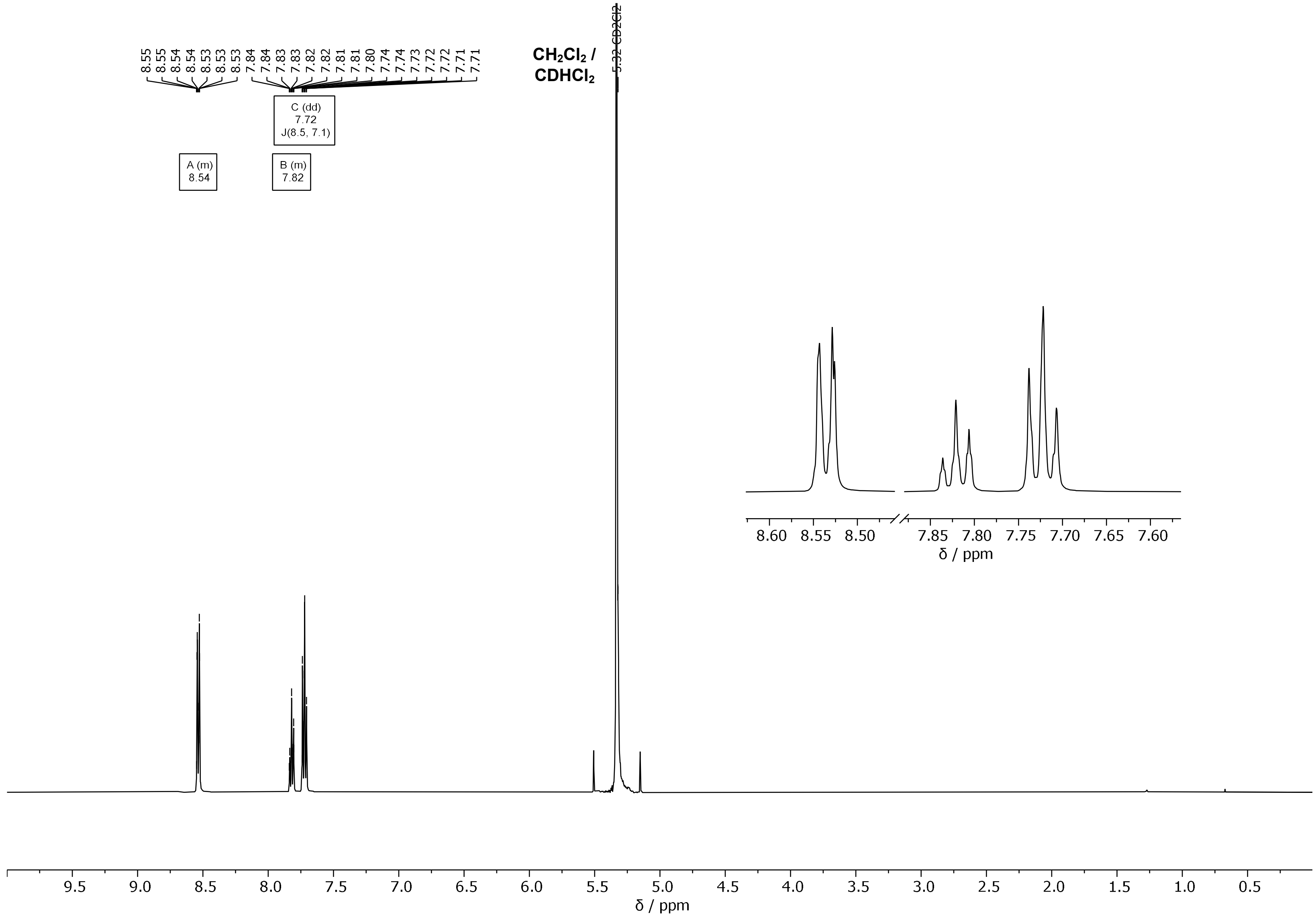

NMR spectrum ***9*****:** ^{1}H NMR spectrum (500 MHz, dichloromethane-d_2) of $TzPh_2$ **3** • BCl_3.

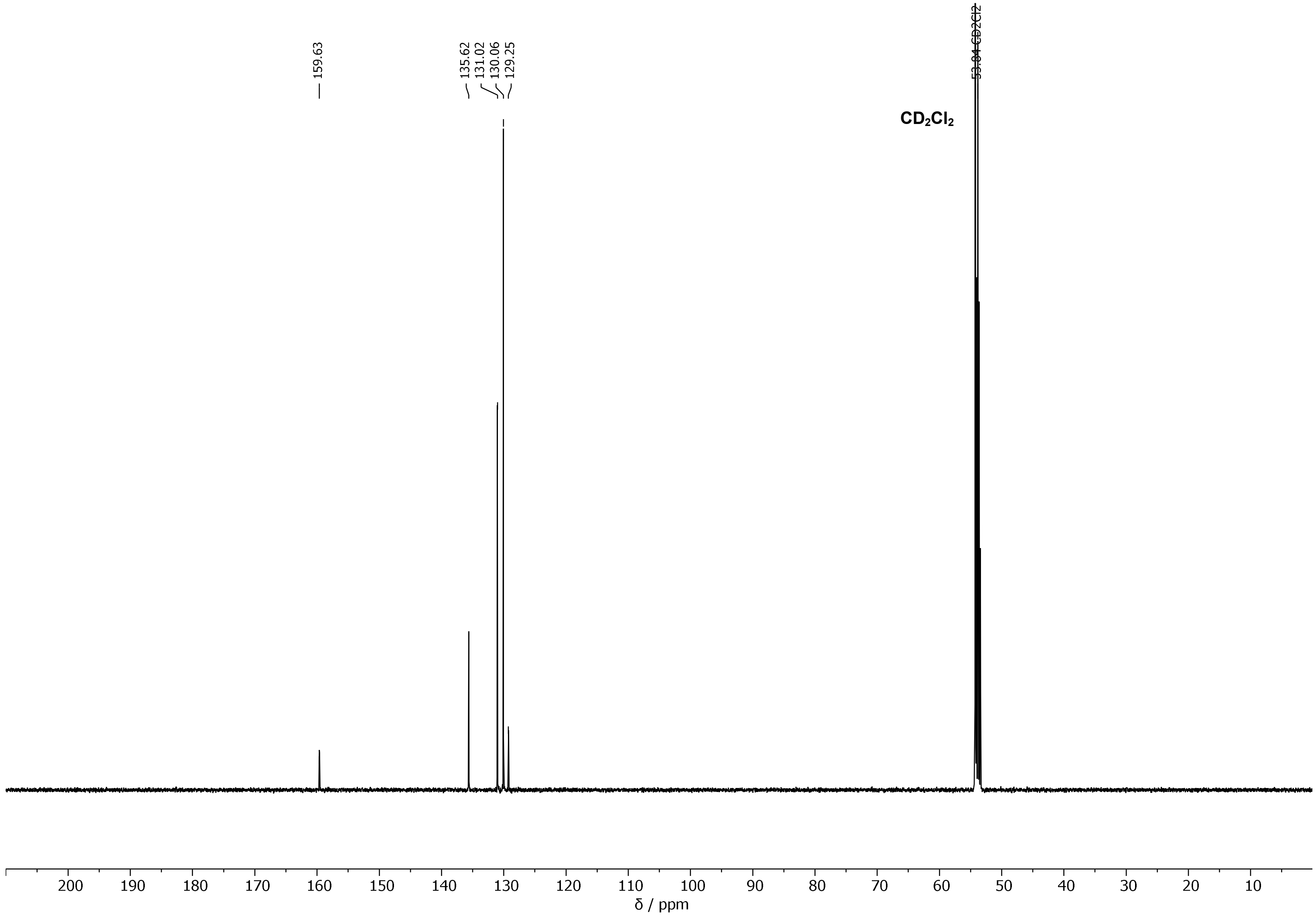

NMR spectrum *10***:** $^{13}C\{^1H\}$ NMR spectrum (126 {500} MHz, dichloromethane-d_2) of $TzPh_2$ **3** • BCl_3.

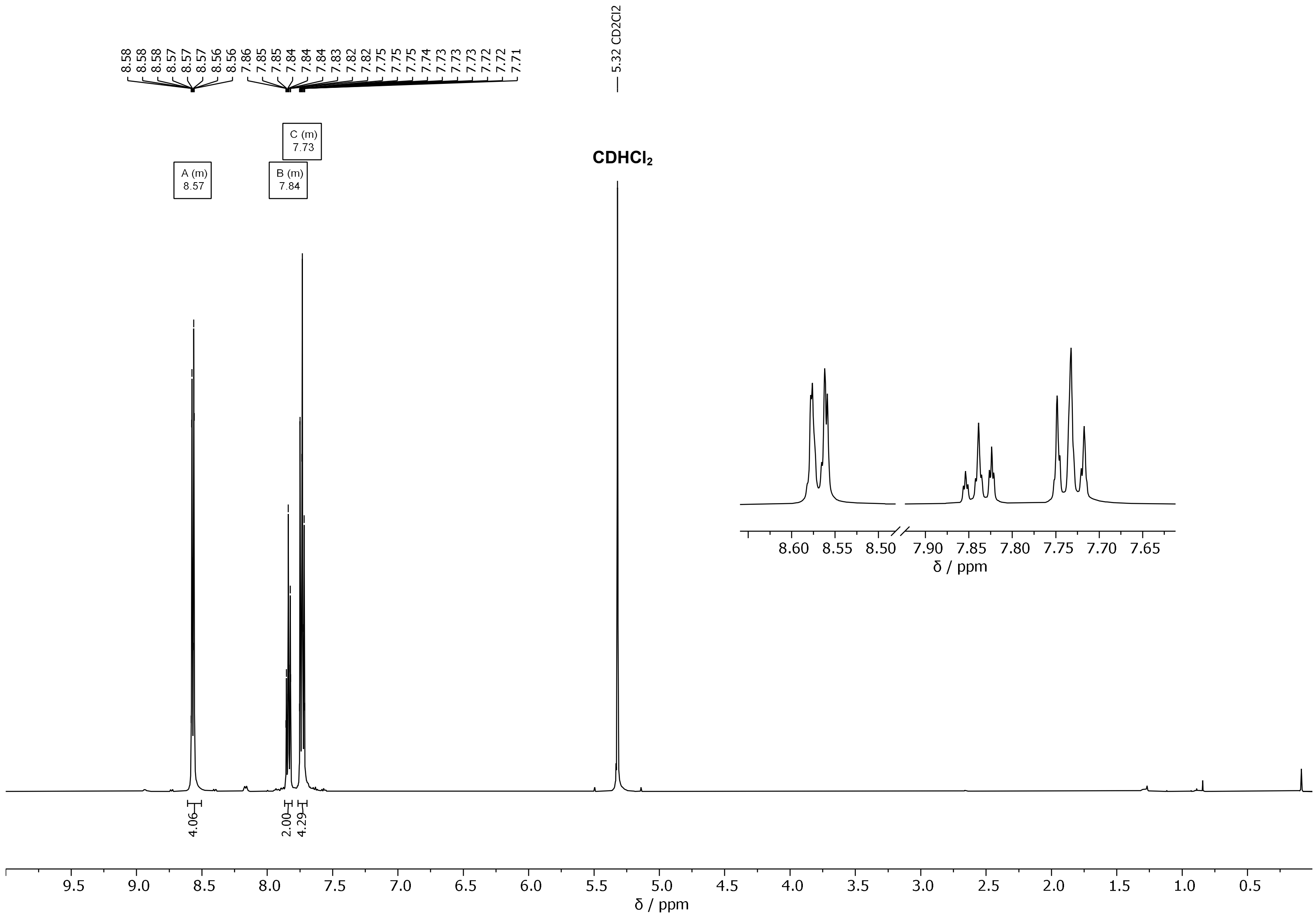

NMR spectrum ***11*****:** ^{1}H NMR spectrum (500 MHz, dichloromethane-d_2) of $TzPh_2$ **3** • BBr_3.

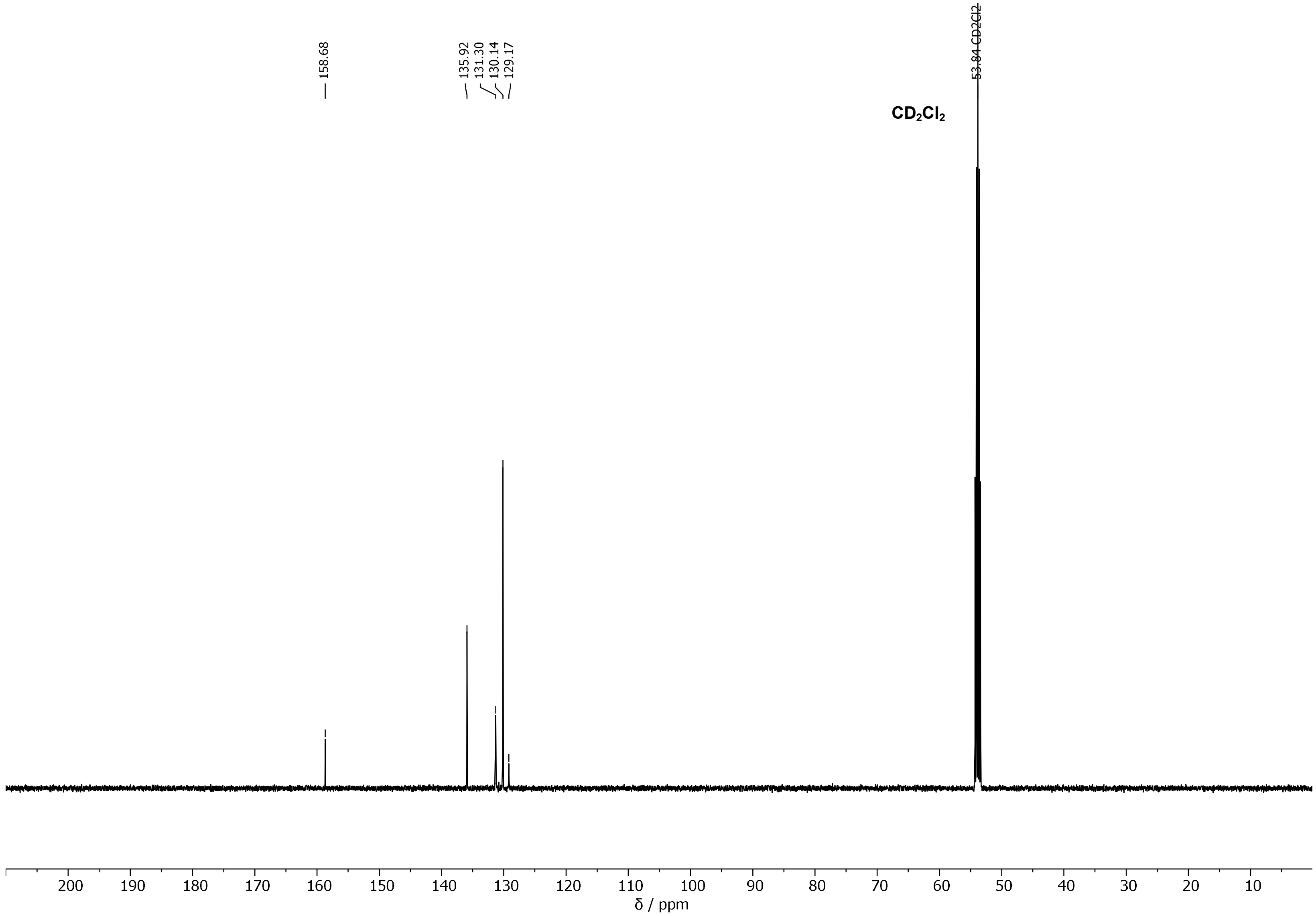

NMR spectrum *12***:** $^{13}C\{^{1}H\}$ NMR spectrum (126 {500} MHz, dichloromethane-d_2) of $TzPh_2$ **3** • BBr_3.

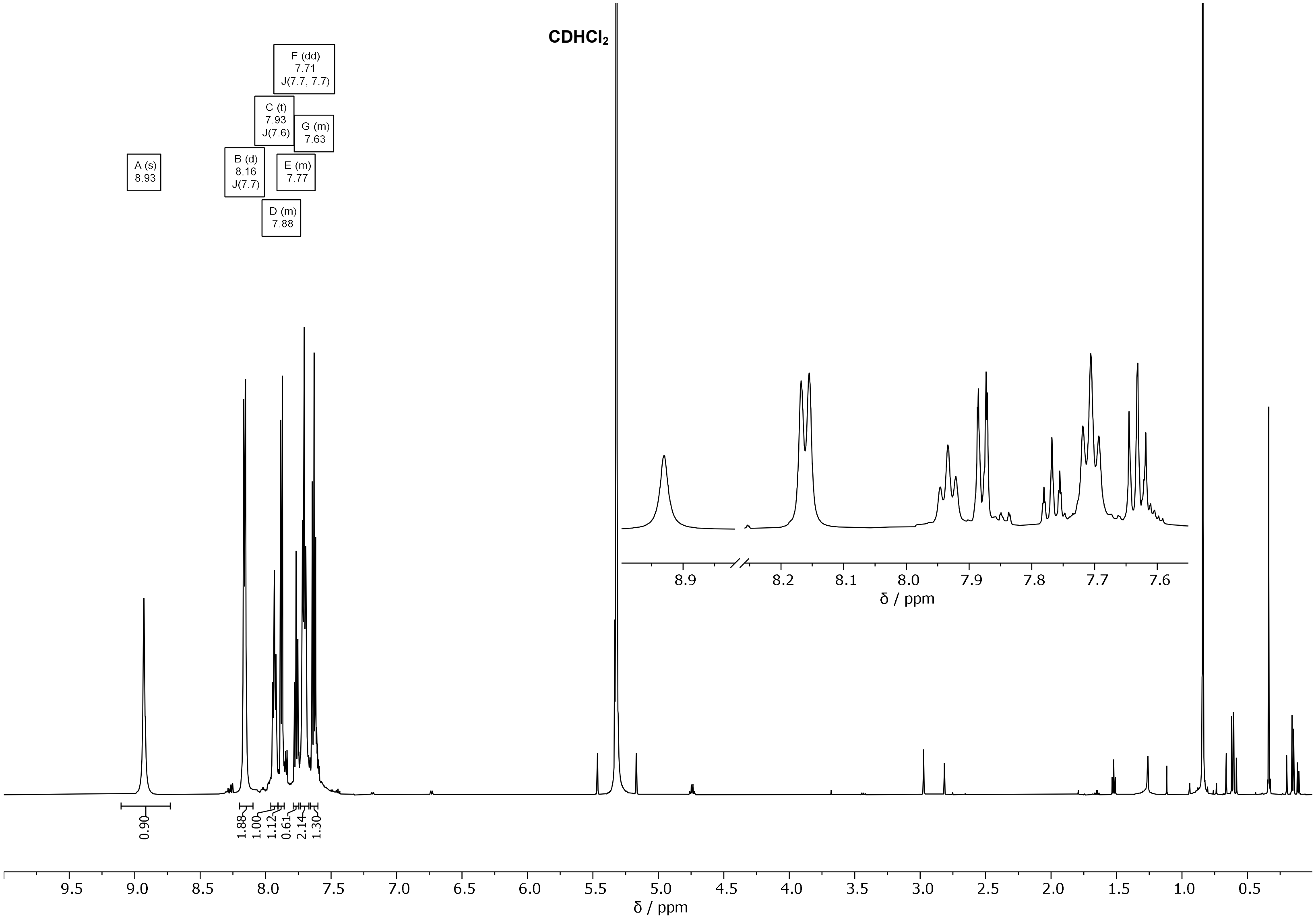

NMR spectrum *13***:** ^{1}H NMR spectrum (600 MHz, dichloromethane-d_2) of a mixture of *d*TzPh$_2$ **2** with BBr_3 (2.5 eq.).

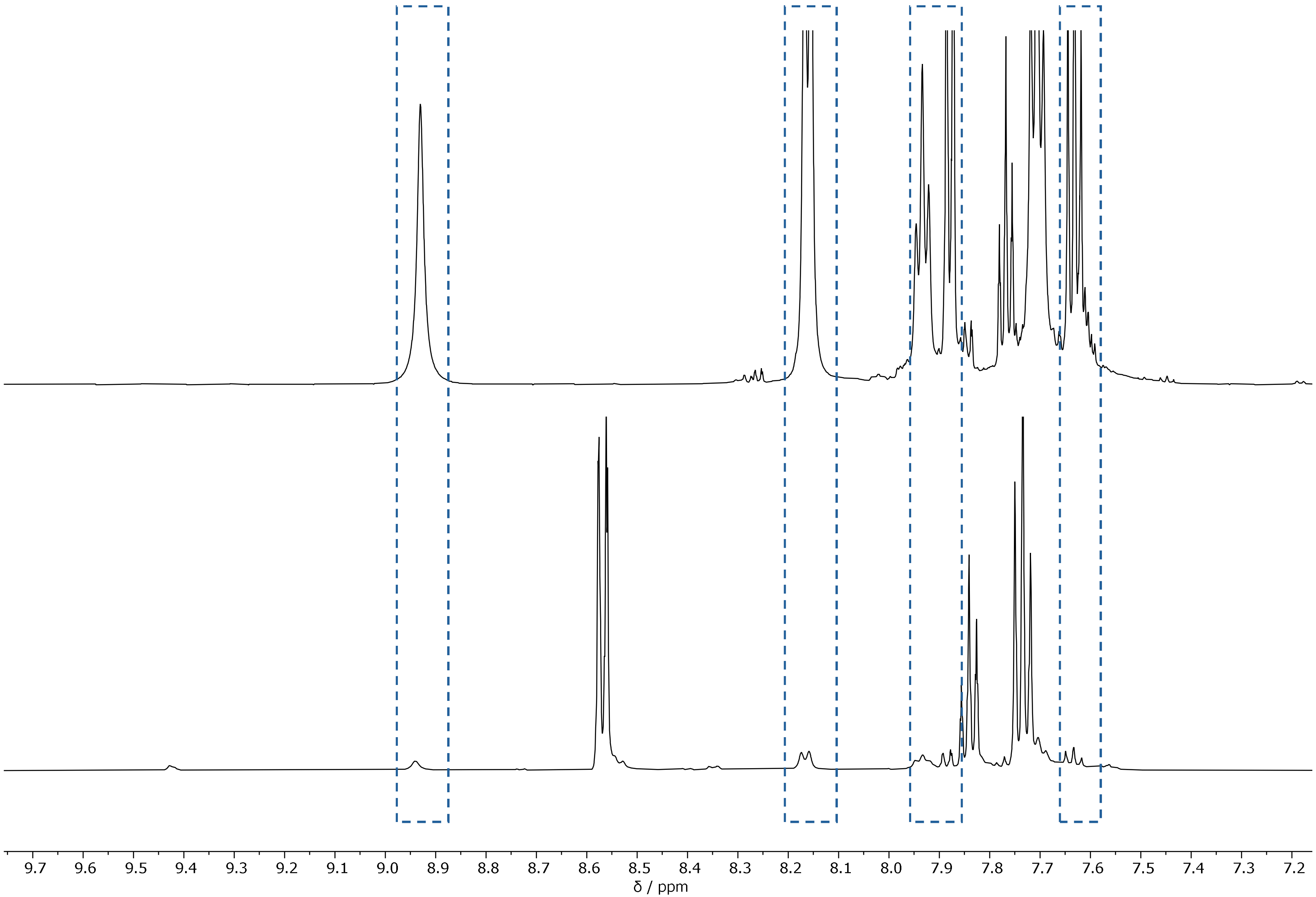

NMR spectrum *14***:** Comparison of ^{1}H NMR spectra (500 MHz, dichloromethane-d_2) of a mixture of *d*TzPh$_2$ **2** with BBr_3 (2.5 eq., top) and a solution of TzPh$_2$ **3** with BBr_3 (2.5 eq., bottom) after three days at room temperature. Dotted boxes indicate signals attributable to *d*TzPh$_2$ **2** • n BBr_3.

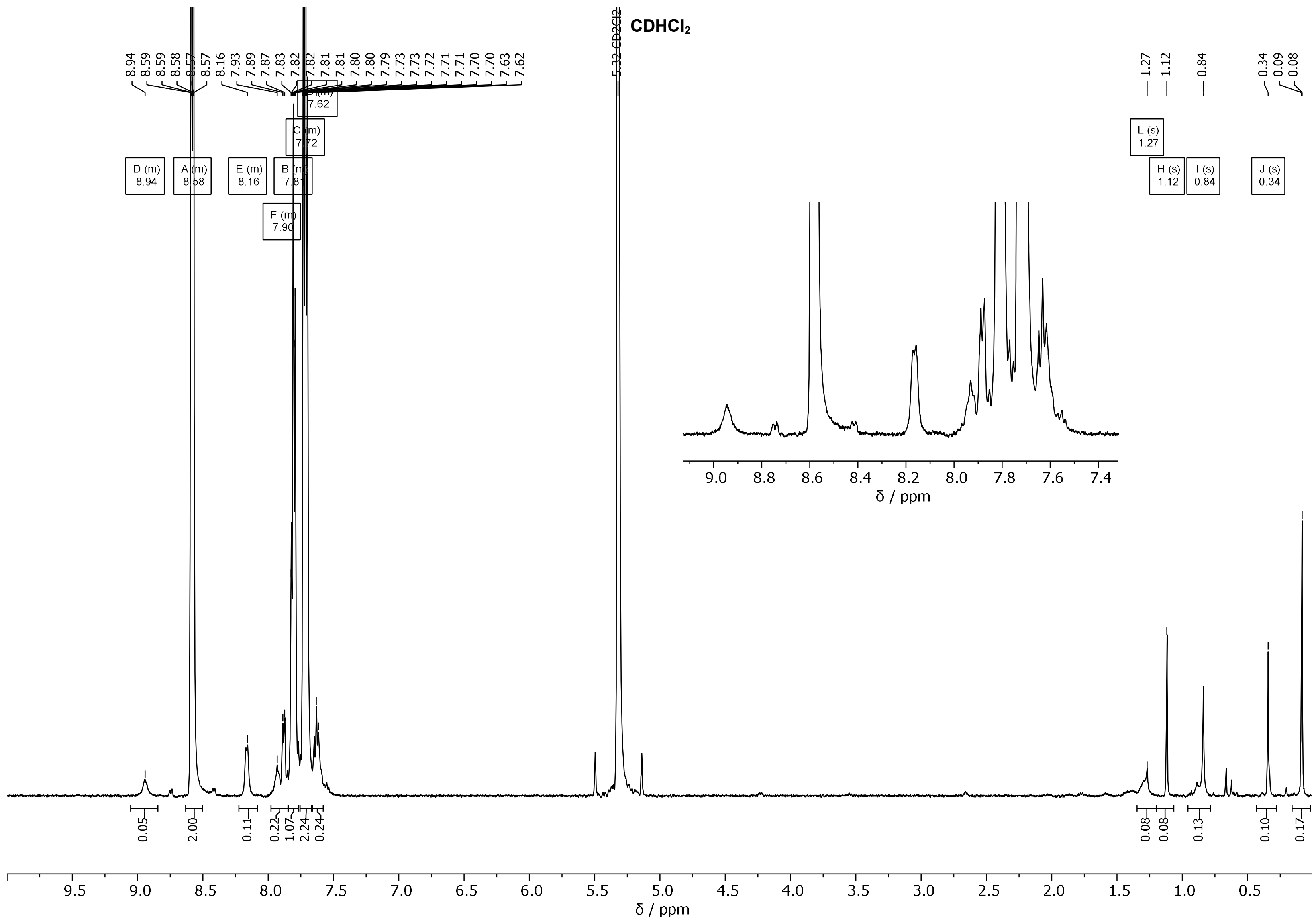

NMR spectrum *15*: Exemplary ^{1}H NMR spectrum (500 MHz, dichloromethane-d_2) of a mixture of TzPh$_2$ **3** with BBr_3 (1.0 eq.) after removal of the solvents with ~5% of decomposition products.

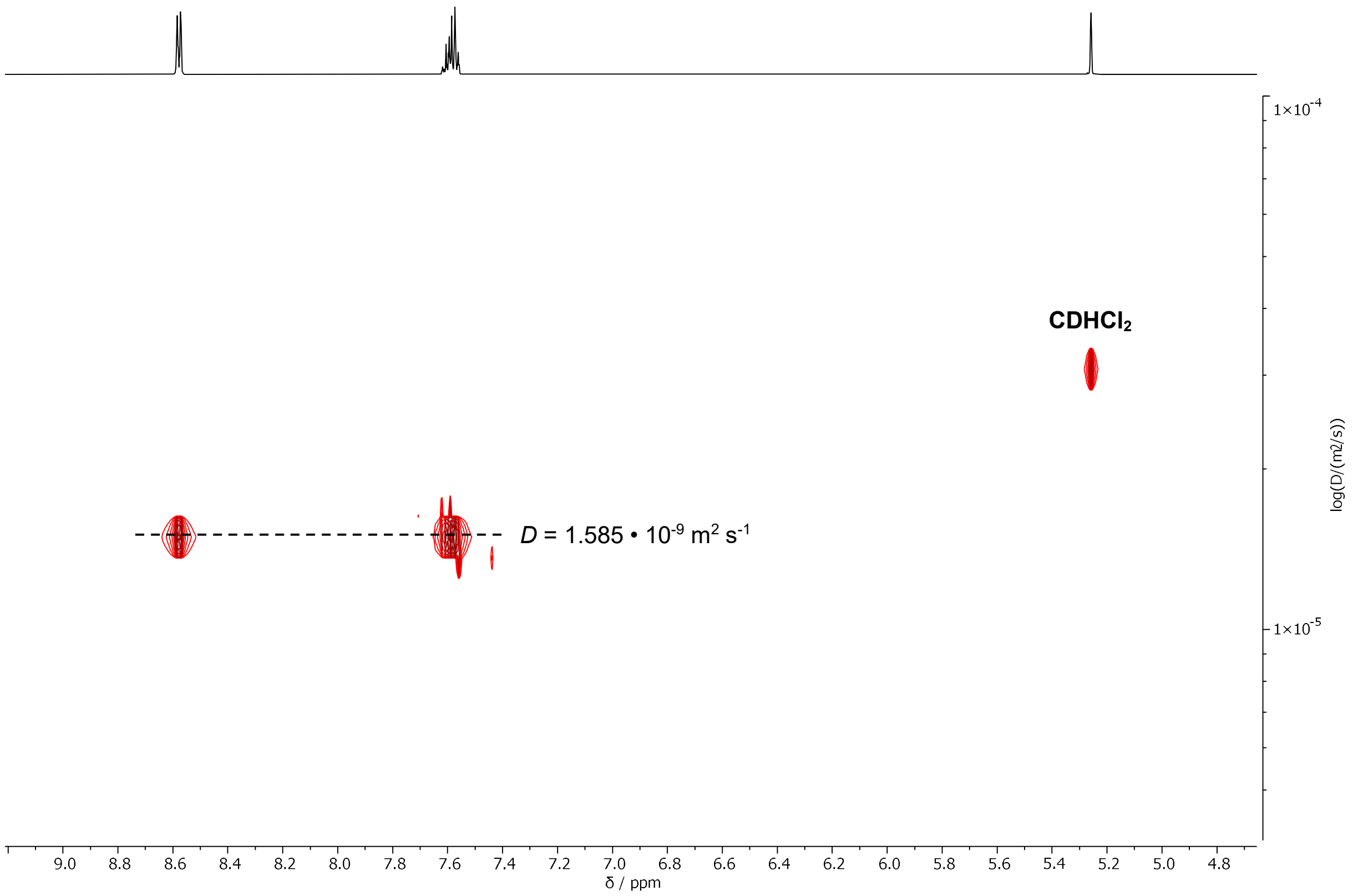

NMR spectrum *16*: ^{1}H DOSY NMR spectrum (600 MHz, dichlormethane-*d*$_2$) of TzPh$_2$ **3** with uncorrected diffusion constant.

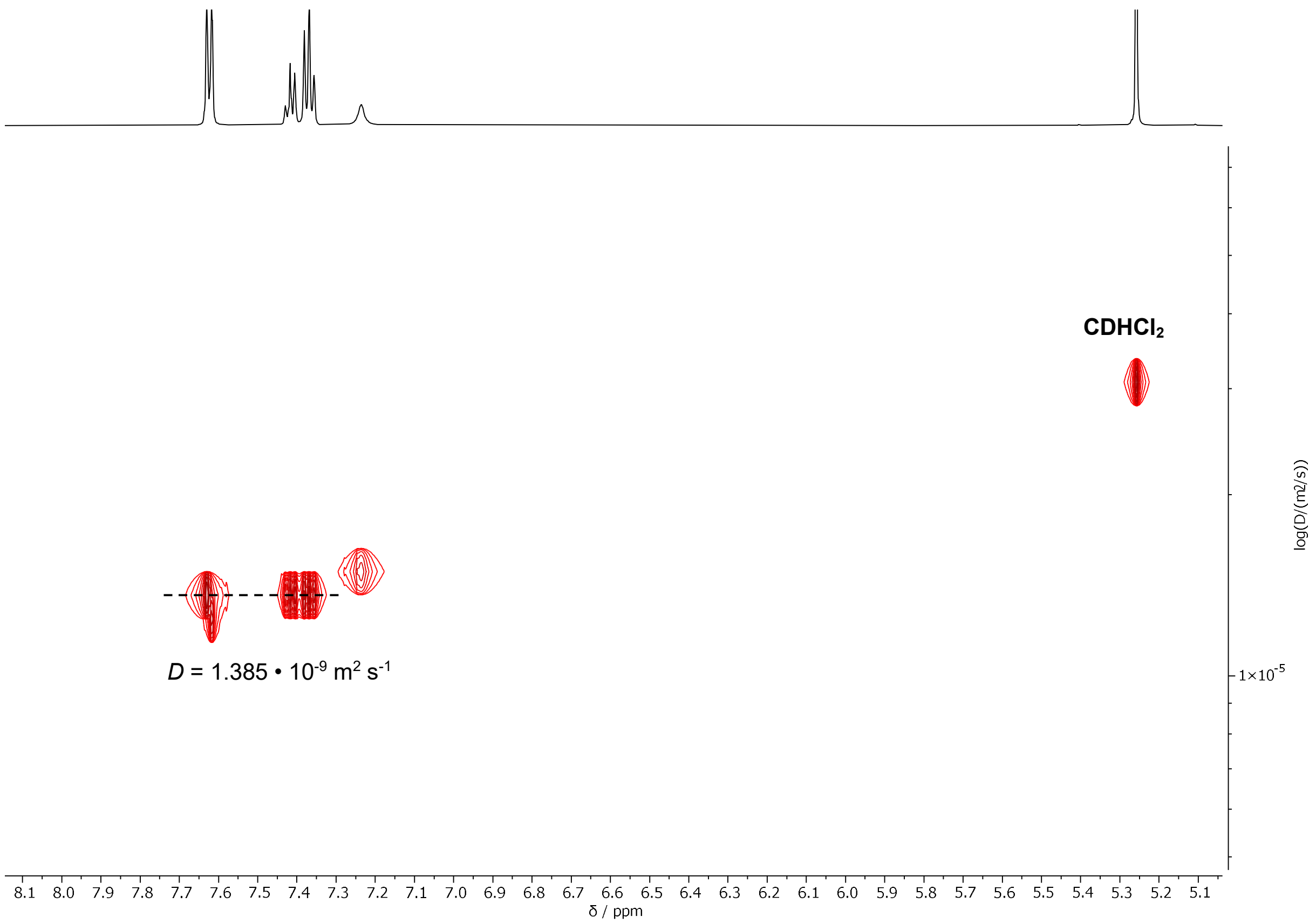

NMR spectrum *17*: ^{1}H DOSY NMR spectrum (600 MHz, dichlormethane-*d*$_2$) of *d*TzPh$_2$ **2** with uncorrected diffusion constant.

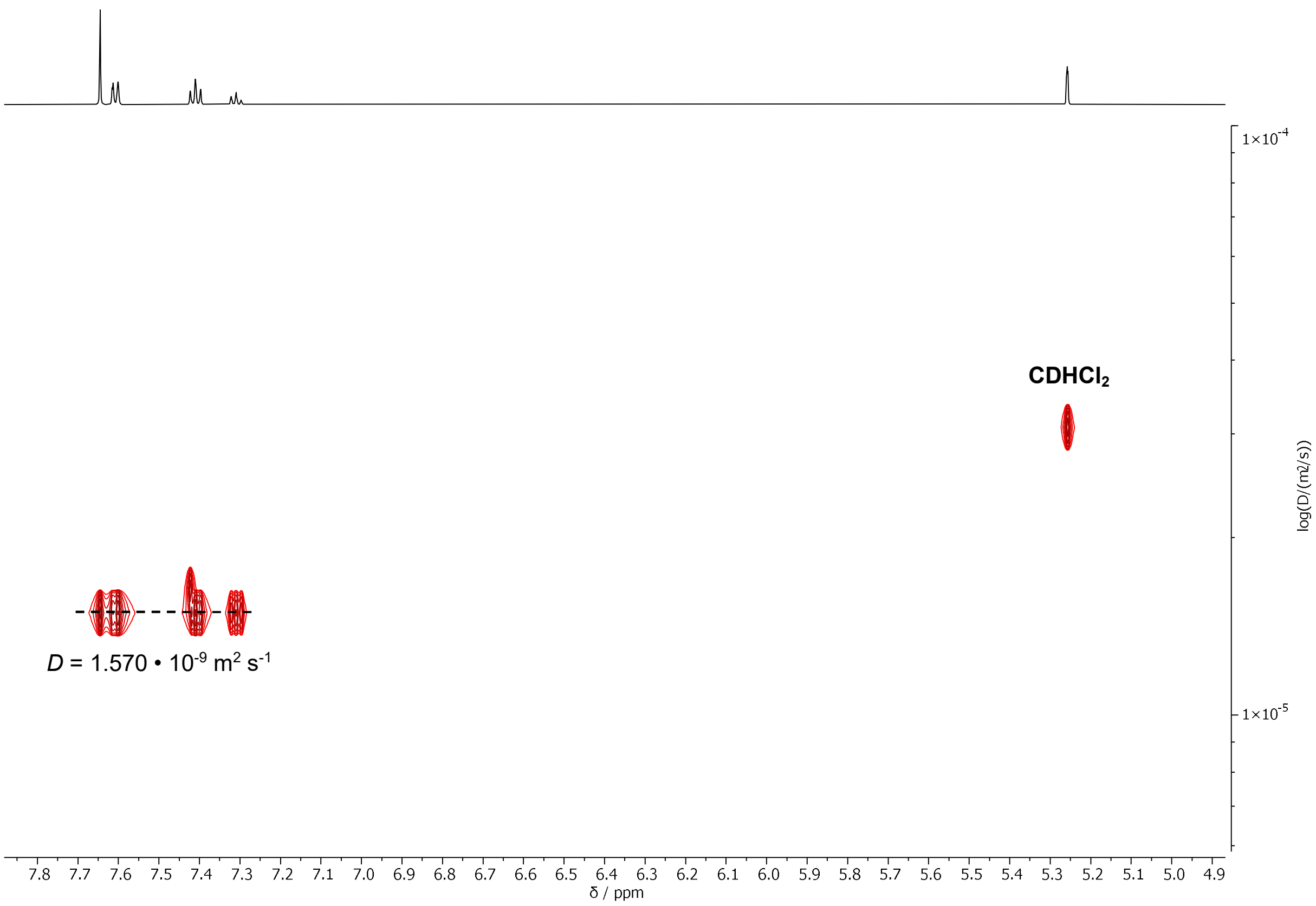

NMR spectrum ***18*****:** ^{1}H DOSY NMR spectrum (600 MHz, dichlormethane-d_2) of *p*-terphenyl with uncorrected diffusion constant.

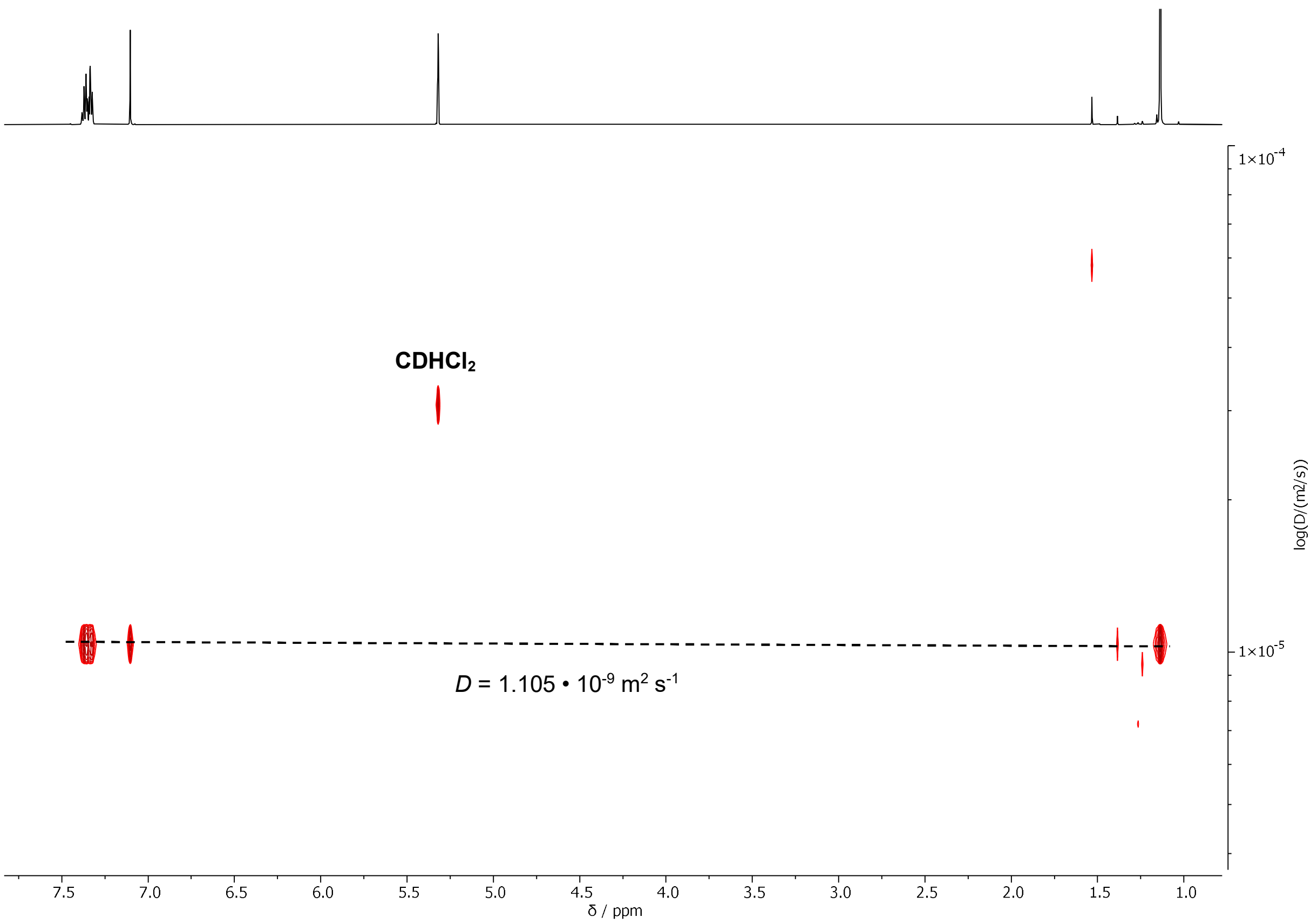

NMR spectrum ***19*****:** ^{1}H DOSY NMR spectrum (600 MHz, dichlormethane-d_2) of 2',5'-di-*tert*-butyl-1,1':4',1''-terphenyl with uncorrected diffusion constant.

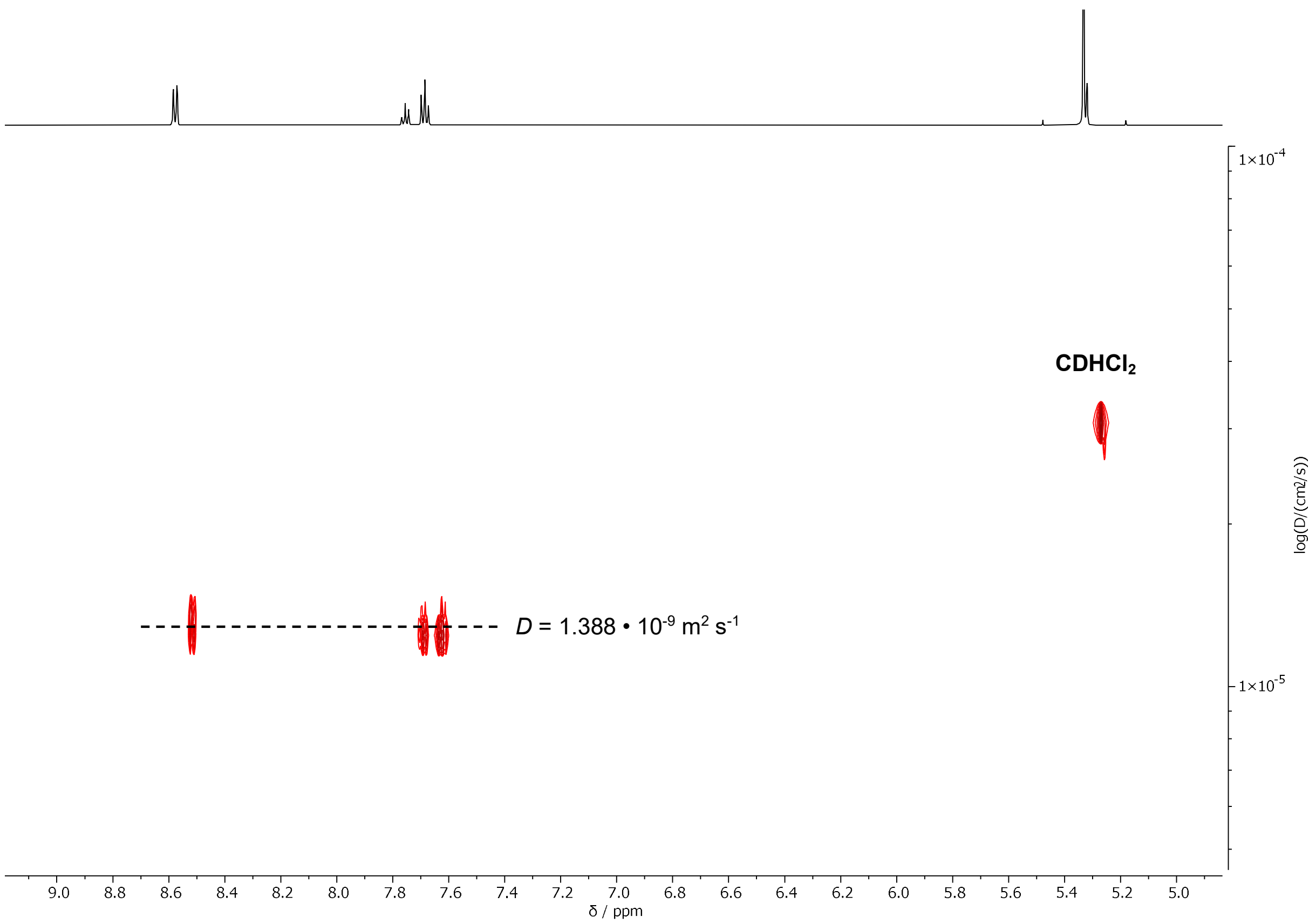

NMR spectrum ***20*****:** Representative ^{1}H DOSY NMR spectrum (600 MHz, dichlormethane-d_2) of a mixture of $TzPh_2$ **3** with BCl_3 (1.0 eq.) with uncorrected diffusion constant.

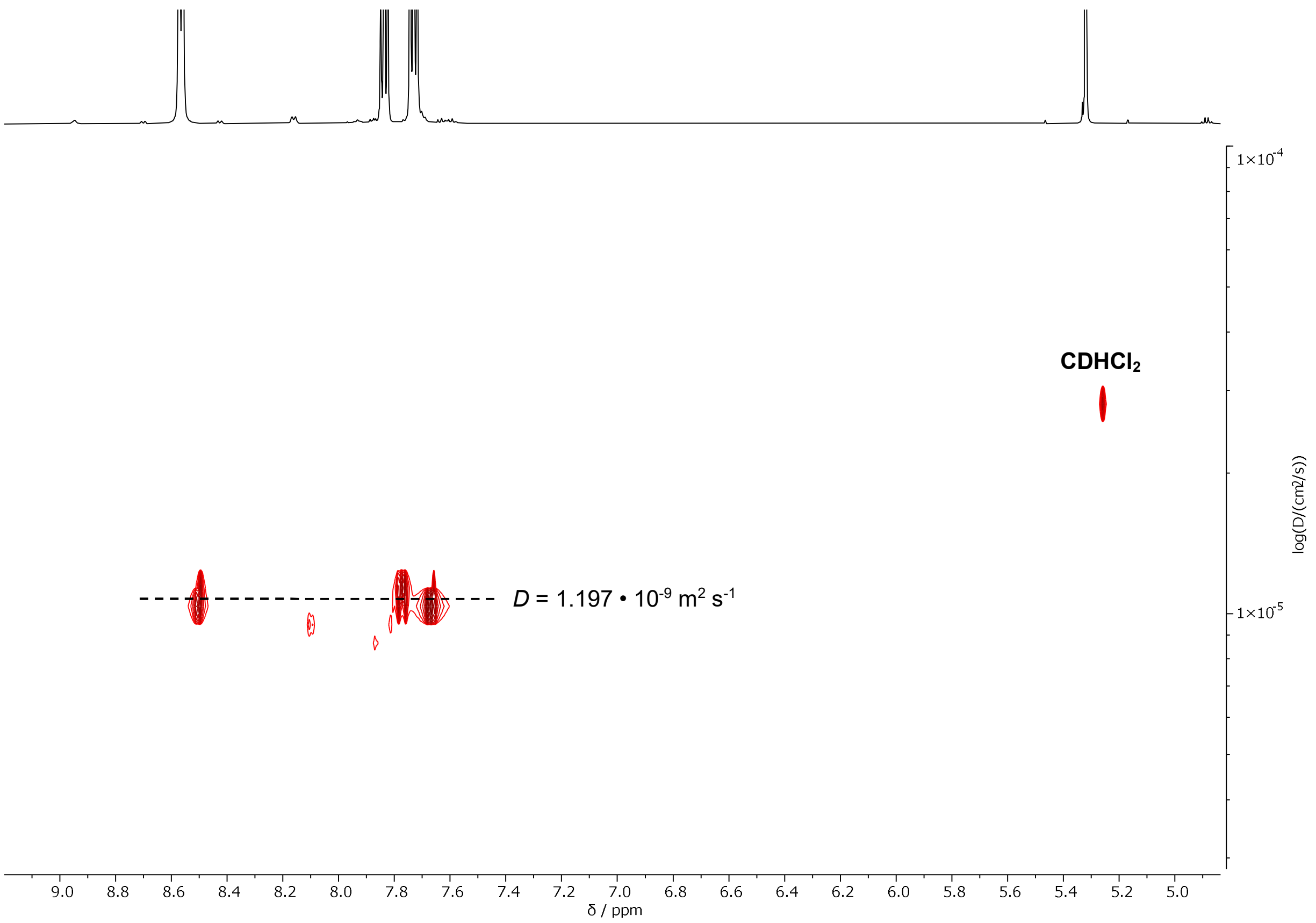

NMR spectrum ***21*****:** Representative ^{1}H DOSY NMR spectrum (600 MHz, dichlormethane-d_2) of a mixture of $TzPh_2$ **3** with BBr_3 (1.0 eq.) with uncorrected diffusion constant.

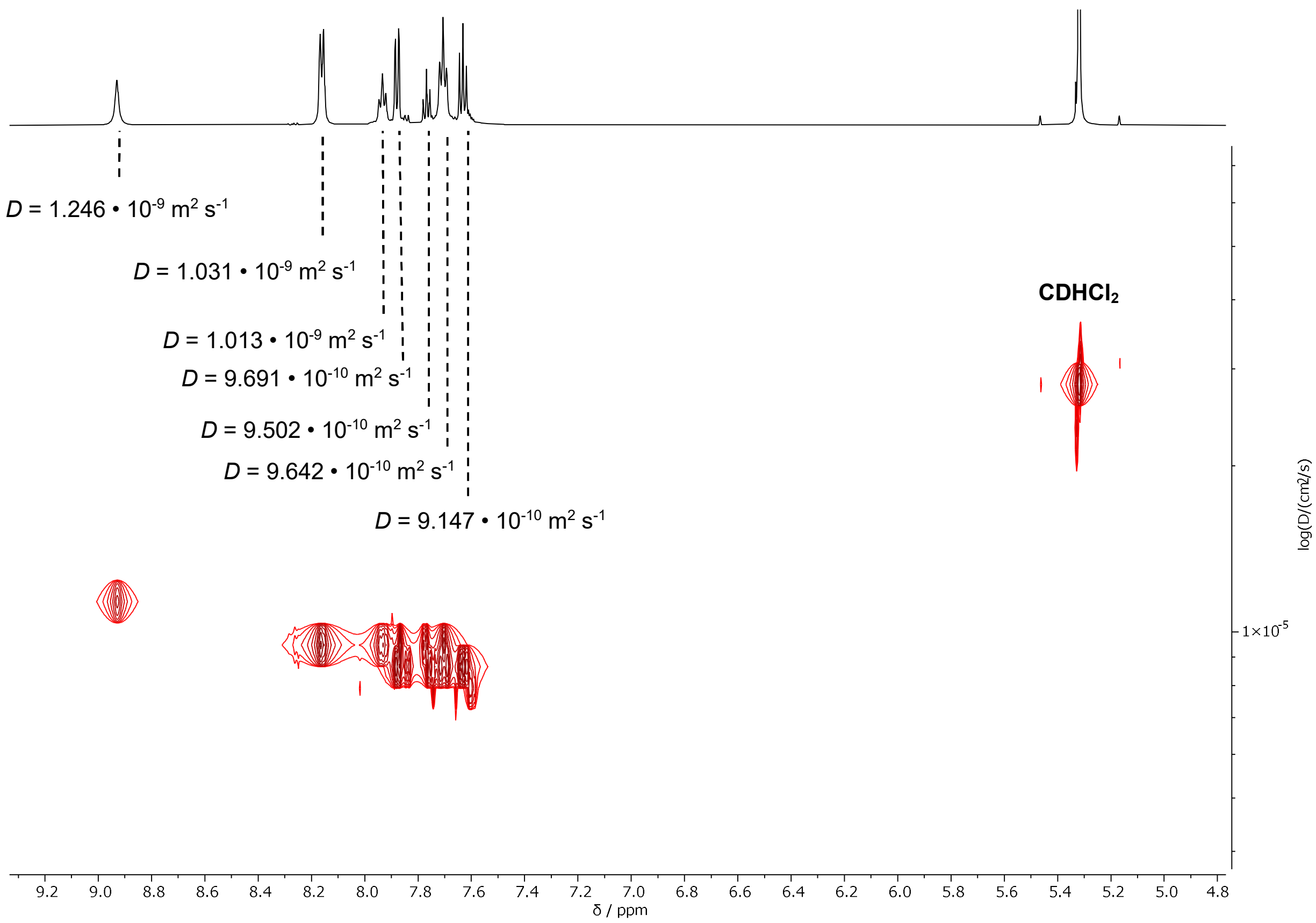

NMR spectrum ***22*****:** ^{1}H DOSY NMR spectrum (600 MHz, dichlormethane-d_2) of a mixture of *d*TzPh$_2$ **2** with BBr_3 (2.5 eq.) with uncorrected diffusion constants.

7 References

[1] G. R. Fulmer, A. J. M. Miller, N. H. Sherden, H. E. Gottlieb, A. Nudelman, B. M. Stoltz, J. E. Bercaw, K. I. Goldberg, *Organometallics* **2010**, *29*, 2176.
[2] O. V. Dolomanov, L. J. Bourhis, R. J. Gildea, J. A. K. Howard, H. Puschmann, *J. Appl. Cryst.* **2009**, *42*, 339.
[3] M. Moral, G. García, A. Peñas, A. Garzón, J. M. Granadino-Roldán, M. Melguizo, M. Fernández-Gómez, *Chem. Phys.* **2012**, *408*, 17.
[4] A. E. Nibbs, A.-L. Baize, R. M. Herter, K. A. Scheidt, *Org. Lett.* **2009**, *11*, 4010.
[5] a) R. Mills, *J. Phys. Chem.* **1973**, *77*, 685; b) W. S. Price, H. Ide, Y. Arata, *J. Phys. Chem. A* **1999**, *103*, 448.
[6] A. Macchioni, G. Ciancaleoni, C. Zuccaccia, D. Zuccaccia, *Chem. Soc. Rev.* **2008**, *37*, 479.
[7] A. Mix, J.-H. Lamm, J. Schwabedissen, E. Gebel, H.-G. Stammler, N. W. Mitzel, *Chem. Commun.* **2022**, *58*, 3465.
[8] Lide, D. R. (Eds.) *"Physical Constants of Organic Compounds" in CRC Handbook of Chemistry and Physics, Internet Version*, **2005**.
[9] T. W. Phillips, K. P. Murphy, *J. Chem. Eng. Data* **1970**, *15*, 304.
[10] H. C. Chen, S. H. Chen, *J. Phys. Chem.* **1984**, *88*, 5118.
[11] Y. H. Zhao, M. H. Abraham, A. M. Zissimos, *J. Org. Chem.* **2003**, *68*, 7368.
[12] S. Alvarez, *Dalton Trans.* **2013**, *42*, 8617.
[13] G. M. Sheldrick, *Acta Crystallogr., Sect. A* **2015**, *71*, 3.
[14] L. J. Bourhis, O. V. Dolomanov, R. J. Gildea, J. A. K. Howard, H. Puschmann, *Acta Crystallogr., Sect. A* **2015**, *71*, 59.
[15] F. Kleemiss, O. V. Dolomanov, M. Bodensteiner, N. Peyerimhoff, L. Midgley, L. J. Bourhis, A. Genoni, L. A. Malaspina, D. Jayatilaka, J. L. Spencer, F. White, B. Grundkötter-Stock, S. Steinhauer, D. Lentz, H. Puschmann, S. Grabowsky, *Chem. Sci.* **2020**, *12*, 1675.
[16] G. M. Sheldrick, *Acta Crystallogr., Sect. C* **2015**, *71*, 3.
[17] K. F. Dziubek, A. Katrusiak, *J. Phys. Chem. B* **2008**, *112*, 12001.
[18] V. R. Thalladi, H.-C. Weiss, D. Bläser, R. Boese, A. Nangia, G. R. Desiraju, *J. Am. Chem. Soc.* **1998**, *120*, 8702.
[19] R. Ranjbar-Karimi, *CCDC 1580478: Experimental Crystal Structure Determination*, Cambridge Crystallographic Data Centre, **2017**.
[20] A. P. Rice, F. S. Tham, E. L. Chronister, *J. Chem. Crystallogr.* **2013**, *43*, 14.
[21] G. Santiso-Quiñones, I. Krossing, *Z. Anorg. Allg. Chem.* **2008**, *634*, 704.
